# Molecular and Cellular Mechanisms for Trapping and Activating Emotional Memories

**DOI:** 10.1371/journal.pone.0161655

**Published:** 2016-08-31

**Authors:** Thomas Rogerson, Balaji Jayaprakash, Denise J. Cai, Yoshitake Sano, Yong-Seok Lee, Yu Zhou, Pallavi Bekal, Karl Deisseroth, Alcino J. Silva

**Affiliations:** 1 Integrative Center for Learning and Memory, Departments of Neurobiology, Psychology, and Psychiatry & Biobehavioral Sciences, and Brain Research Institute, Los Angeles, California, United States of America; 2 Department of Bioengineering, Psychiatry and Behavioral Sciences, Neurosciences Program, CNC Program, Howard Hughes Medical Institute, Stanford University, Stanford, California, United States of America; State University of New York Downstate Medical Center, UNITED STATES

## Abstract

Recent findings suggest that memory allocation to specific neurons (i.e., neuronal allocation) in the amygdala is not random, but rather the transcription factor cAMP-response element binding protein (CREB) modulates this process, perhaps by regulating the transcription of channels that control neuronal excitability. Here, optogenetic studies in the mouse lateral amygdala (LA) were used to demonstrate that CREB and neuronal excitability regulate which neurons encode an emotional memory. To test the role of CREB in memory allocation, we overexpressed CREB in the lateral amygdala to recruit the encoding of an auditory-fear conditioning (AFC) memory to a subset of neurons. Then, post-training activation of these neurons with Channelrhodopsin-2 was sufficient to trigger recall of the memory for AFC, suggesting that CREB regulates memory allocation. To test the role of neuronal excitability in memory allocation, we used a step function opsin (SFO) to transiently increase neuronal excitability in a subset of LA neurons during AFC. Post-training activation of these neurons with Volvox Channelrhodopsin-1 was able to trigger recall of that memory. Importantly, our studies show that activation of the SFO did not affect AFC by either increasing anxiety or by strengthening the unconditioned stimulus. Our findings strongly support the hypothesis that CREB regulates memory allocation by modulating neuronal excitability.

## Introduction

There is growing evidence for molecular and cellular mechanisms that regulate the allocation of memories to specific synapses and neurons in a neurocircuit [1]. By directing related information to overlapping populations of neurons, memory allocation mechanisms are thought to link these memories, place them within a common context, save storage space and alter memory strength. Memory allocation mechanisms may also organize the storage of information into component elements that encode features shared across related experiences, thereby linking the storage of these experiences [1, 2]. Previous results suggest that the transcription factor cAMP-response element binding protein (CREB) has a role in neuronal allocation in the lateral amygdala (LA) [3–9], a structure required for learning the association between a tone (conditioned stimulus or CS) and foot-shock (unconditioned stimulus or US) in auditory-fear conditioning (AFC) [10–12]. Increasing the levels of CREB in a subset of LA neurons increased the probability that these neurons were engaged in AFC [3]. Silencing or ablating these neurons triggered deficits in recall [4, 5]. Knowing that these neurons are necessary for recall, we tested whether post-training optogenetic activation of these neurons is sufficient to elicit recall in the absence of external cues. To test this hypothesis we used lentiviral vectors to increase the levels of CREB in a subset of LA neurons. Following training in AFC, Channelrhodopsin-2 (ChR2) was used to activate these neurons with virally encoded CREB. This activation triggered recall of the AFC memory, a result further supporting a role for CREB in memory allocation.

Recently, CREB has been proposed to regulate neuronal allocation by modulating neuronal excitability [5, 6, 9]. Importantly, electrophysiological studies indicated that LA neurons with higher CREB levels are more excitable [5, 13]. Here, we tested whether it is possible to recruit the encoding of an auditory-fear conditioning (AFC) memory to a subset of neurons with increased excitability, as previously shown with CREB. To test this hypothesis we used a step function opsin (SFO) to transiently increase excitability in a subset of LA neurons during AFC, and show that post-training activation of these neurons with Volvox Channelrhodopsin-1 can also trigger recall of that memory. Our findings strongly support the hypothesis that CREB regulates memory allocation by modulating neuronal excitability.

## Results

### CREB and neuronal allocation in the lateral amygdala

**Fig 1:**
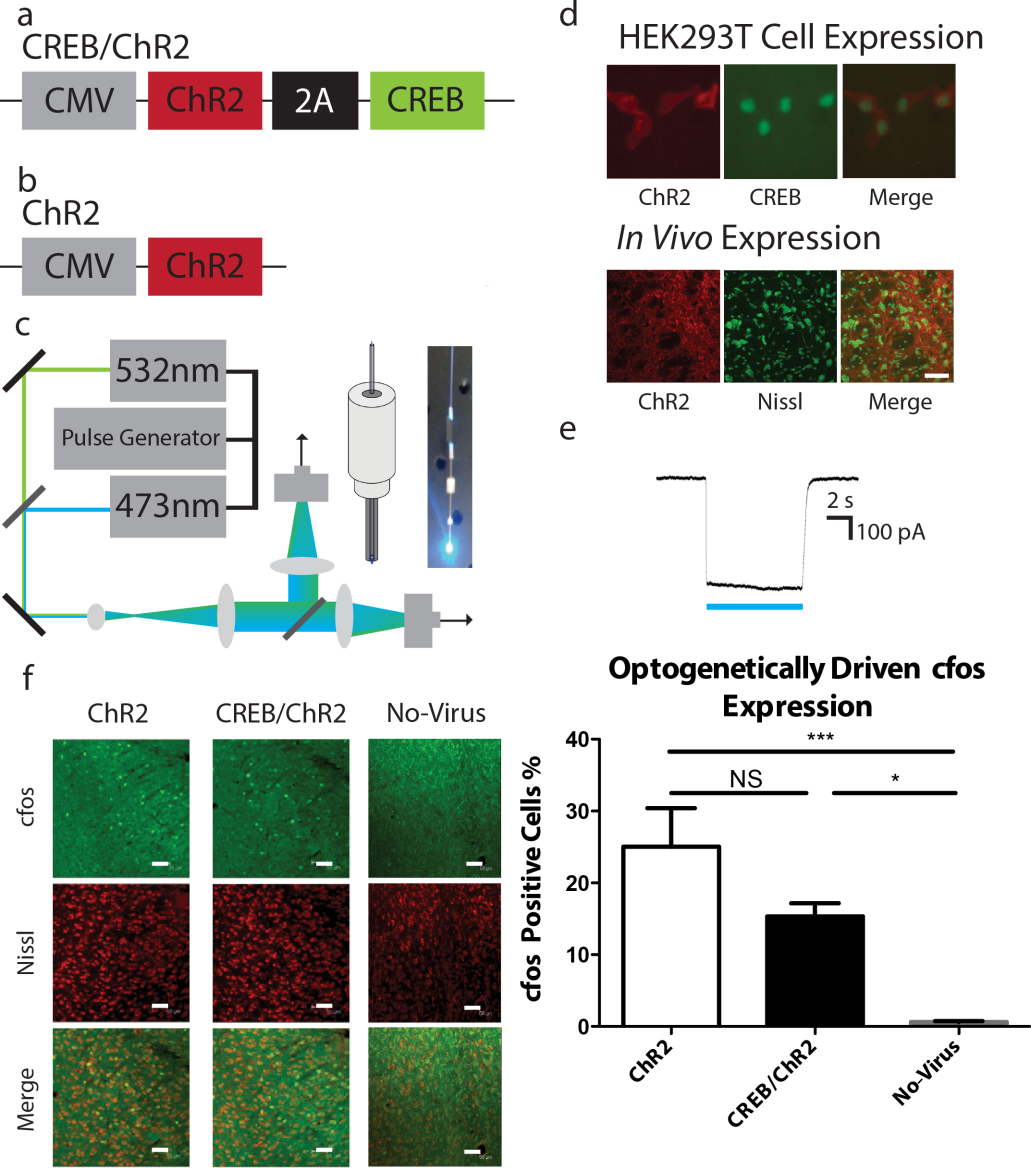
Viral design, optogenetic setup, expression, electrophysiological and molecular characterization of CREB/ChR2 and ChR2. **(a)** CREB/ChR2, **(b)** ChR2 and **(c)** Optogenetics setup. **(d)** Expression of CREB/ChR2 in HEK293T cells and *in vivo*. HEK293T cells were infected with CREB/ChR2 and the expression of CREB and ChR2 was visualized by EGFP and mCherry fluorescence respectively. Cells *in vivo* were stained for mCherry and are counterstained with Nissl. Scale bar, 50um. **(e)** Whole-cell recordings from CREB/ChR2 expressing HEK293T. Inward depolarizing currents were observed during 473nm light illumination. **(f)** The LA of CREB/ChR2 (n = 5) and ChR2 (n = 7) expressing animals was illuminated with 473nm light while another cohort of control animals also received 473nm light illumination, but expressed no opsin (No-Virus, n = 6). The animals were sacrificed and their brains were stained for cfos. There was a significant difference in cfos expression between cohorts (one-way ANOVA, F(2,15) = 11.46, p < 0.001). Tukey’s HSD post hoc test determined CREB/ChR2 (p < 0.05) and ChR2 (p < 0.001) cohorts had a higher proportion of cfos positive neurons compared to the No-Virus cohort. The percent of cfos positive cells represents the fraction of cfos positive neurons over the total number of neurons as counted by nissl staining. ChR2; 25.05 ± 5.36%, CREB/ChR2; 15.33 ± 1.83%, No-Virus; 0.58 ± 0.14%. Error bars are mean ± SEM, * = p < 0.05, *** = p < 0.001, NS = not significant.

We increased CREB levels in a subset of LA neurons with a lentivirus expressing a CREB gene. The memory allocation hypothesis [6, 7] predicts that these neurons would be preferentially chosen to encode a memory for AFC. This lentivirus (CREB/ChR2; **Fig 1A**) also included a ChR2 gene [14] that allows for the specific activation of the LA neurons expressing the viral CREB gene. As a control, we used a virus that only expresses ChR2 (ChR2; **Fig 1B**). ChR2 is a light-gated ion channel that is activated by blue (473nm) light. To illuminate the infected neurons and manipulate their activity in behaving mice, an optogenetics setup was created to bilaterally deliver light to the LA through optical fibers (**Fig 1C**). We confirmed that under physiological conditions, activation of ChR2 can activate LA neurons (**Fig 1E and 1F**). Therefore, if the LA neurons infected with viral CREB are more likely to encode a memory for AFC, then activation of these neurons with blue light should trigger stronger recall than activation of LA neurons infected with the control virus.

To confirm expression of the virus in the amygdala, immunocytochemistry studies with an antibody against mCherry, a marker present in the two lentiviruses described above, detected a region 250um from the tip of the injector infected by the CREB/ChR2 and ChR2 viruses (**Fig 1D**). To confirm that ChR2 was functioning as expected in our viral constructs, HEK293T cells were infected with CREB/ChR2 (**Fig 1D**). Then, whole-cell recordings were carried out with and without 473nm illumination (**Fig 1E**). As expected inward depolarizing currents were observed that lasted for the duration of the 473nm light pulse. To confirm that ChR2 was working as expected *in vivo*, the LA was infected with either CREB/ChR2 or ChR2. A control cohort of animals was not infected with either virus (No-Virus). Subsequently, the three cohorts: CREB/ChR2, ChR2 and No-Virus had their LA illuminated with 473nm light. Ninety minutes following the illumination, the mice were sacrificed and their brains were stained for cfos, a common marker of neuronal activity [15]. The CREB/ChR2 and ChR2 cohorts had a larger population of cfos positive neurons than the No-Virus cohort (Fig 1F), thereby demonstrating that ChR2 in both constructs is capable of activating LA neurons.

**Fig 2:**
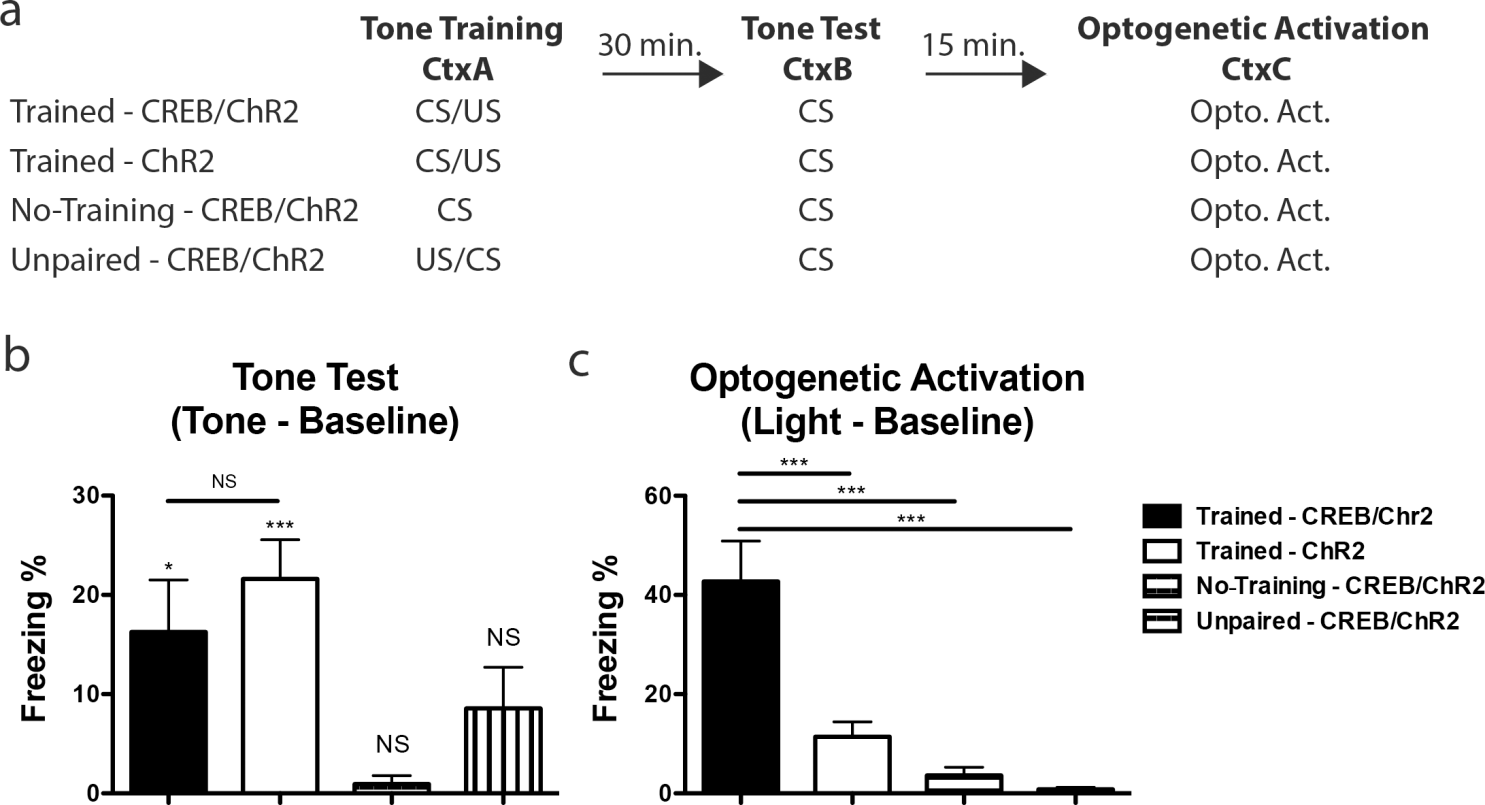
Optogenetic activation of a CREB allocated memory trace. **(a)** Behavioral design for the four experimental cohorts: Trained—CREB/ChR2, Trained—ChR2, No-Training–CREB/ChR2 and Unpaired—CREB/ChR2. **(b)** There was a difference in freezing between cohorts during the tone test (one-way ANOVA, F(3,45) = 5.51, p < 0.01). However, Tukey’s HSD post hoc test determined that there was no difference between Trained—CREB/ChR2 and the Trained—ChR2 cohorts (p > 0.05). One-sample t-tests against 0 demonstrate that both the Trained—CREB/ChR2 and the Trained—ChR2 cohorts significantly acquired the auditory-fear memory (t(9) = 3.11, p < 0.05 and t(16) = 5.48, p < 0.001) whereas the No-Training—CREB/ChR2 and the Unpaired—CREB/ChR2 cohorts did not (t(10) = 1.09, p > 0.05 and t(10) = 2.06, p > 0.05). Mean freezing levels were: Trained—CREB/ChR2 16.27 ± 5.24%, n = 10; Trained—ChR2 21.62 ± 3.95%, n = 17; No-Training—CREB/ChR2 0.93 ± 0.85%, n = 11; and Unpaired—CREB/ChR2 8.56 ± 4.16%, n = 11. **(c)** There was a difference in freezing between cohorts during the optogenetic activation (one-way ANOVA, F(3,43) = 18.19, p < 0.001). Tukey’s HSD post hoc test determined that Trained—CREB/ChR2 froze significantly more than Trained—ChR2 (p < 0.001), No-Training—CREB/ChR2 (p < 0.001) and Unpaired—CREB/ChR2 cohorts (p < 0.001). Mean freezing levels were: Trained—CREB/ChR2 42.73 ± 8.17%, n = 11; Trained—ChR2 11.44 ± 2.98%, n = 15; No-Training—CREB/ChR2 3.64 ± 1.63%, n = 11; and Unpaired—CREB/ChR2 0.83 ± 0.37%, n = 10. Error bars are mean ± SEM, * = p < 0.05, *** = p < 0.001, NS = not significant. See S1 Fig for baseline freezing levels and analysis.

We observed the recall of an AFC memory during post-training optogenetic activation of virally CREB expressing LA neurons, an observation consistent with the hypothesis that overexpression of CREB biases the allocation of the AFC memory towards those cells. This was achieved in the Trained—CREB/ChR2 cohort of mice by bi-laterally infecting a sub-population of LA neurons with CREB/ChR2 prior to AFC (Tone Training). Following AFC their memory was tested (Tone Test), 30-minutes later, by playing the conditioning tone in a novel chamber. Fifteen minutes after confirmation that these mice showed tone conditioning, they were placed in a third context, and 473nm light was delivered to the LA for 1 min (Optogenetic Activation) (**Fig 2A**). Both the tone test and optogenetic activation were completed within 60 minutes of training, so as to avoid confounding CREB’s role on memory allocation with its well-known role in memory consolidation [16]. CREB is known not to affect memory 60 minutes after training [16]. Although freezing was apparent in the Trained—CREB/ChR2 cohort upon optogenetic activation, it is possible that optogenetic activation of any similar sized subpopulation of LA neurons is sufficient to elicit the recall of a previously encoded AFC memory. To test for this possibility, we compared the freezing levels elicited by optogenetic activation of virally CREB expressing neurons (Trained—CREB/ChR2) to a similar population not expressing CREB (Trained—ChR2). Unlike the Trained—CREB/ChR2 the Trained—ChR2 cohort had a sub-population of their LA neurons infected with ChR2 alone. Importantly, both cohorts were given AFC (Tone Training), tone tested (Tone Test) and optogenetically activated (Optogenetic Activation) identically (**Fig 2A**).

As expected, there was no difference in freezing to tone between the Trained—CREB/ChR2 and the Trained—ChR2 cohorts, demonstrating that both groups learned the tone-shock association to similar levels **(Fig 2B)**. This result demonstrates that viral-encoded CREB did not affect the acquisition or retrieval of AFC at this early time point. Optogenetic activation, however, triggered higher freezing in the Trained—CREB/ChR2 than in the Trained—ChR2 cohorts (**Fig 2C**), a result that supports the CREB memory allocation hypothesis.

It is possible, however, that optogenetic activation of a subpopulation of LA neurons virally expressing CREB may be sufficient to elicit freezing even in the absence of AFC training, perhaps due to the additive effects of ChR2 stimulation and CREB-dependent increases in excitability. To control for this possibility, we compared the freezing levels elicited by optogenetic activation of virally CREB expressing neurons in the key experimental cohort (Trained—CREB/ChR2) to two additional control cohorts (No-Training—CREB/ChR2, Unpaired—CREB/ChR2) also infected with CREB/ChR2 but not trained on AFC. These control cohorts were trained as previously described except no US was delivered in the No-Training—CREB/ChR2 cohort and the US was delivered prior to CS presentation in the Unpaired—CREB/ChR2 cohort (**Fig 2A**). As expected, freezing in response to the tone test was not above baseline levels in both the No-Training—CREB/ChR2 and Unpaired—CREB/ChR2 cohorts (**Fig 2B**). Importantly, optogenetic activation of the No-Training—CREB/ChR2 and Unpaired—CREB/ChR2 cohorts failed to trigger recall (**Fig 2C**), demonstrating that the freezing observed in the experimental (Trained—CREB/ChR2) cohort was due to the reactivation of a learned AFC memory and not to additive effects of ChR2 and CREB-dependent increases in excitability.

The results presented above demonstrate that post-training optogenetic activation of LA neurons with virally expressing CREB triggers freezing responses that reflect recall of AFC, a result that supports the hypothesis that increasing the levels of CREB in a subpopulation of LA neurons dramatically enhances the probability that these neurons encode a memory for AFC. A subpopulation of ChR2 infected neurons is expected by chance to be incorporated into the memory trace. During optogenetic activation this chance overlap will lead to the AFC memory trace being partially activated in the Trained—ChR2 cohort, resulting in some freezing. Although optogenetic activation of Trained—ChR2 mice resulted in some freezing, the levels were much lower than those observed for Trained—CREB/ChR2 (**Fig 2C**). The CREB memory allocation hypothesis predicted that unlike neurons infected with CREB/ChR2, the neurons infected with ChR2 are not preferentially chosen to participate in memory for AFC. In this respect, it is important to note that AFC levels (as assessed during the Tone Test) in the Trained—CREB/ChR2 and Trained—ChR2 cohorts were indistinguishable, a result that demonstrates that the differences in freezing between these two cohorts during optogenetic activation are not due to differences in AFC learning, but reflect instead the proposed bias of memory allocation towards neurons that express high levels of CREB.

### Excitability and the modulation of neuronal allocation

**Fig 3:**
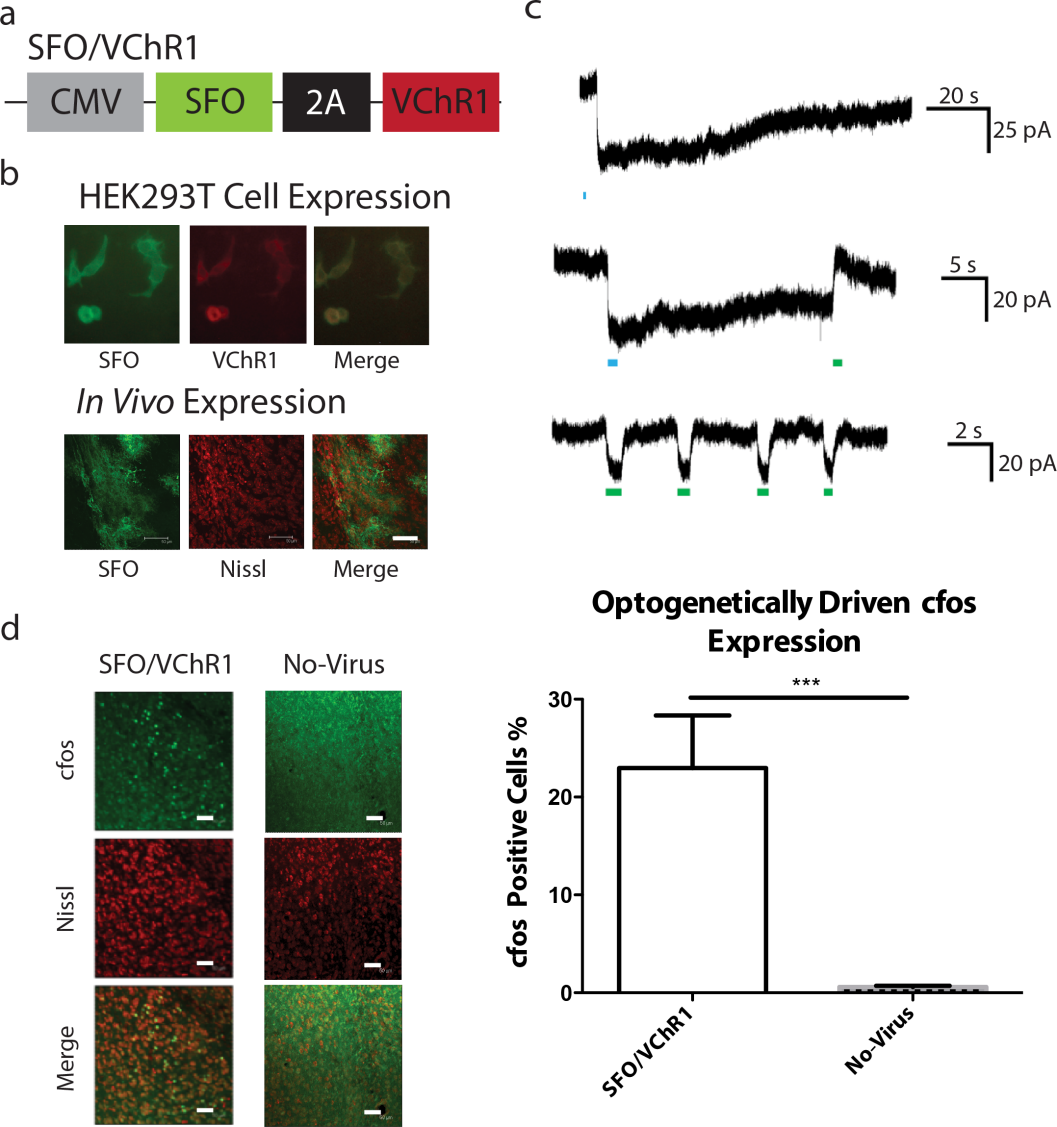
Viral design, expression, and electrophysiological and molecular characterization of SFO/VChR1. **(a)** SFO/VChR1 viral design. **(b)** Expression of SFO/VChR1 in HEK293T cells and *in vivo*. HEK293T cells were infected with SFO/VChR1; VChR1 and SFO were visualized by mCherry and EYFP fluorescence respectively. Cells *in vivo* were stained for EYFP and were counterstained with Nissl. Scale bar, 50um. **(c)** Whole-cell recordings from SFO/VChR1 expressing HEK293T. A 473nm light pulse, designed to activate SFO, triggered a stable depolarizing step in membrane potential that was maintained following the offset of the pulse and reversed by a 532nm green light pulse. Additionally, inward depolarizing currents were generated that lasted for the duration of a 532nm green light pulses designed to turn on VChR1. **(d)** The LA of one cohort of SFO/VChR1 (n = 4) expressing animals was illuminated with 532nm green light while another cohort of control animals also received 532nm light illumination but did not express an opsin (No-Virus, n = 6). The animals were sacrificed and the brains were stained for cfos. The SFO/VChR1 cohort had a significantly larger population of cfos positive neurons than the No-Virus cohort (unpaired, two-sided t-test, t(8) = 5.25, p < 0.001). The percent of cfos positive cells represent the fraction of cfos positive neurons over the total number of neurons as counted by nissl staining: SFO/VChR1-Light; 22.96 ± 5.38%, No Virus; 0.58 ± 0.14%. Error bars are mean ± SEM, *** = p < 0.001.

Previous studies have shown that increasing the CREB levels of LA neurons results in an increase in their excitability [5, 13]. This finding suggested the hypothesis that the increase in neuronal excitability in LA neurons with higher CREB levels is responsible for their preferential inclusion into memory traces [5, 6]. To test the possible involvement of increases in neuronal excitability in memory allocation, we engineered a new viral vector (SFO/VChR1; **Fig 3A**) with a step-function opsin (SFO) to modulate excitability [17] and a Volvox Channelrhodopsin-1 (VChR1) vector to optogenetically activate neurons with 532nm light [18]. We did not use ChR2 for optogenetic activation in this vector, because the 473 nm light required to activate ChR2 would have also activated the SFO present in the vector (**Fig 3A**). We first confirmed that optogenetic activation of SFO with 473 nm blue light leads to prolonged depolarization following the offset of light, and that optogenetic activation of VChR1 with 532 nm light results in amygdala activation *in vivo* (**Fig 3C and 3D**). Immunocytochemistry studies with a EYFP antibody detected a region 250um from the tip of the injector infected by SFO/VChR1 (**Fig 3B**).

To confirm that SFO and VChR1 were functioning as expected in our viral constructs, HEK293T cells were infected with SFO/VChR1 (**Fig 3B**). Then, whole-cell recordings were carried out with and without illumination with 473nm light and (separately) 532nm (green) light (**Fig 3C**). SFO activation resulted in a stable depolarizing step in membrane potential that was maintained following the offset of the 473nm light pulse. We also observed VChR1 generated inward depolarizing currents that lasted for the duration of the 532nm light pulses. To confirm that VChR1 was working as expected *in vivo*, analogous cfos experiments to those performed with CREB/ChR2 and ChR2 were carried out. As expected the SFO/VChR1 cohort revealed a larger population of cfos positive neurons than the No-Virus cohort that received identical light activation. This result demonstrates that VChR1 was capable of activating LA neurons (**Fig 3D**).

**Fig 4:**
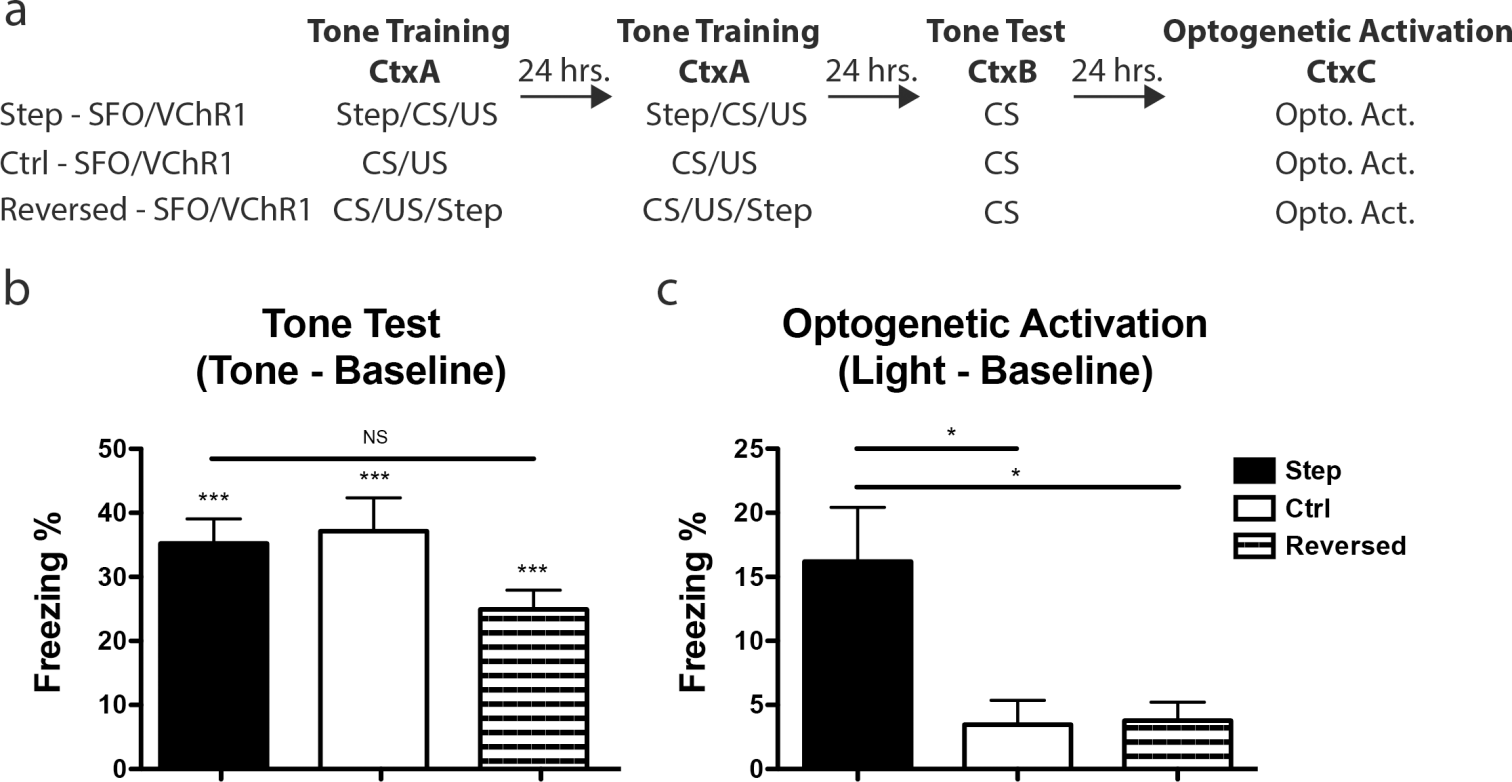
Optogenetic activation of a memory trace allocated by excitability. **(a)** Behavioral design for the three experimental cohorts: Step—SFO/VChR1, Ctrl—SFO/VChR1 and Reversed—SFO/VChR1. **(b)** There is no difference in freezing during tone test between the three cohorts (one-way ANOVA, F(2,46) = 2.56 p > 0.05). The mean freezing for each cohort: Step—SFO/VChR1; 35.21 ± 3.84%, n = 21, Ctrl—SFO/VChR1; 37.13 ± 5.24%, n = 12 and Reversed—SFO/VChR1; 24.92 ± 3.00%, n = 16. One-sample t-test against 0 demonstrate that the Step—SFO/VChR1 (t(20) = 9.17, p < 0.001), Ctrl—SFO/VChR1 (t(11) = 7.09, p < 0.001) and Reversed—SFO/VChR1 (t(15) = 8.31, p < 0.001) cohorts froze above baseline levels. **(c)** There was a significant difference in freezing during optogenetic activation (one-way ANOVA, F(2,45) = 4.94, p < 0.05). Tukey’s HSD post hoc test determined that Step—SFO/VChR1 cohort froze more during optogenetic activation than both Ctrl—SFO/VChR1 (p < 0.05) and Reversed-SFO/VChR1 (p < 0.05) cohorts. The mean freezing was calculated for Step—SFO/VChR1; 16.19 ± 4.25%, n = 21, Ctrl—SFO/VChR1; 3.47 ± 1.90%, n = 12, and Reversed—SFO/VChR1 cohort; 3.78 ± 1.43%, n = 15. Error bars are mean ± SEM, * = p < 0.05, *** = p < 0.001. See S2 Fig -for baseline freezing levels and analysis.

To test whether an increase in excitability biases the allocation of an AFC memory, a sub-population of LA neurons was transfected with SFO/VChR1 prior to training. The resulting mice were trained with two trials of AFC separated by 24 hours and received a 5-second-long pulse of 473nm light *prior* to each AFC trial. Please note that the mice were trained with two trials of AFC to compensate for the fact that mice tethered (with fiber optics) during training demonstrate reduced learning compared to non-tethered mice. One day after the last AFC training session, the mice were given a tone test in a novel environment. The following day the same mice were placed in a third context and underwent optogenetic activation for 1 minute as described above, except that this time we used 532nm light to activate VChR1 (**Fig 4A**). Although optogenetic activation, triggered an increase in freezing in the mice transfected with the Step–SFO/VChR1 vector, it is possible that optogenetic activation of any similar sized subpopulation of LA neurons is sufficient to elicit the recall of a previously encoded AFC memory. To test for this possibility, we compared the freezing levels elicited by optogenetic activation of neurons whose excitability had been increased at the time of learning (Step–SFO/VChR1) to a similar population whose excitability had been not been increased at the time of learning (Ctrl–SFO/VChR1). Like the Step–SFO/VChR1 the Ctrl–SFO/VChR1 cohort had a sub-population of their LA neurons infected with SFO/VChR1 and was trained with two trials of AFC separated by 24 hours. However, the Ctrl—SFO/VChR1 cohort received only sham illumination (no light) during AFC whereas the Step—SFO/VChR1 cohort received a 5-second-long pulse of 473nm light *prior* to each AFC trial as described above. Importantly, following differential training both cohorts were tone tested (Tone Test) and optogenetically activated (Optogenetic Activation) identically (**Fig 4A**).

As expected, there was no difference in freezing to tone between the Step—SFO/VChR1 and the Ctrl—SFO/VChR1 mice, demonstrating that both groups learned the tone-shock association to similar levels **(Fig 4B)**. Although both cohorts showed similar levels of tone-triggered memory, optogenetic activation revealed higher freezing in the Step—SFO/VChR1 cohort compared to the Ctrl—SFO/VChR1 (**Fig 4C**). Please note that the freezing levels triggered by optogenetic activation with VChR1 were lower than those triggered with ChR2; this may be due to the known lower efficiency of VChR1 [18, 19]. These results support the hypothesis that neuronal excitability plays a role in the allocation of memory to specific neurons in a circuit: the results presented above show that optogenetic activation of neurons whose excitability had been artificially increased with SFO at the time of learning were more likely to be engaged in memory than control neurons.

It is possible, however, that optogenetic activation of a subpopulation of LA neurons together with a non-specific increase in excitability (e.g., an increase in excitability after learning) is sufficient to elicit higher freezing, potentially due to the effects of increases in excitability on memory consolidation [9]. To test this possibility, we compared the freezing levels triggered by optogenetic activation of neurons whose excitability had been increased at the time of learning (Step—SFO/VChR1) to an additional control in which excitability was increased immediately after learning (Reversed—SFO/VChR1). The Reversed—SFO/VChR1 cohort was infected with SFO/VChR1 and trained identically to the Step—SFO/VChR1 cohort, except it received a pulse of 473 nm light *following* the tone/shock presentation on each AFC trial (**Fig 4A**). As detailed above, the Step—SFO/VChR1 cohort received the 5 second pulse of 473 nm light immediately *before* training, so as to trigger an increase in excitability in a subset of neurons during training. As expected, freezing in response to a tone test was indistinguishable between the Step—SFO/VChR1 and Reversed—SFO/VChR1 cohorts (**Fig 4B**). Importantly, unlike optogenetic activation of the Step—SFO/VChR1 cohort, optogenetic activation of the Reversed—SFO/VChR1 cohort failed to elicit recall (**Fig 4C**), demonstrating that the freezing observed in the key experimental (Step—SFO/VChR1) cohort was not due to enhancements in memory consolidation caused by increases in excitability. These results strongly support the hypothesis that increases in excitability prior to encoding are a key mechanism of memory allocation in the LA. These results also show that increases in excitability following training do not affect memory allocation.

**Fig 5:**
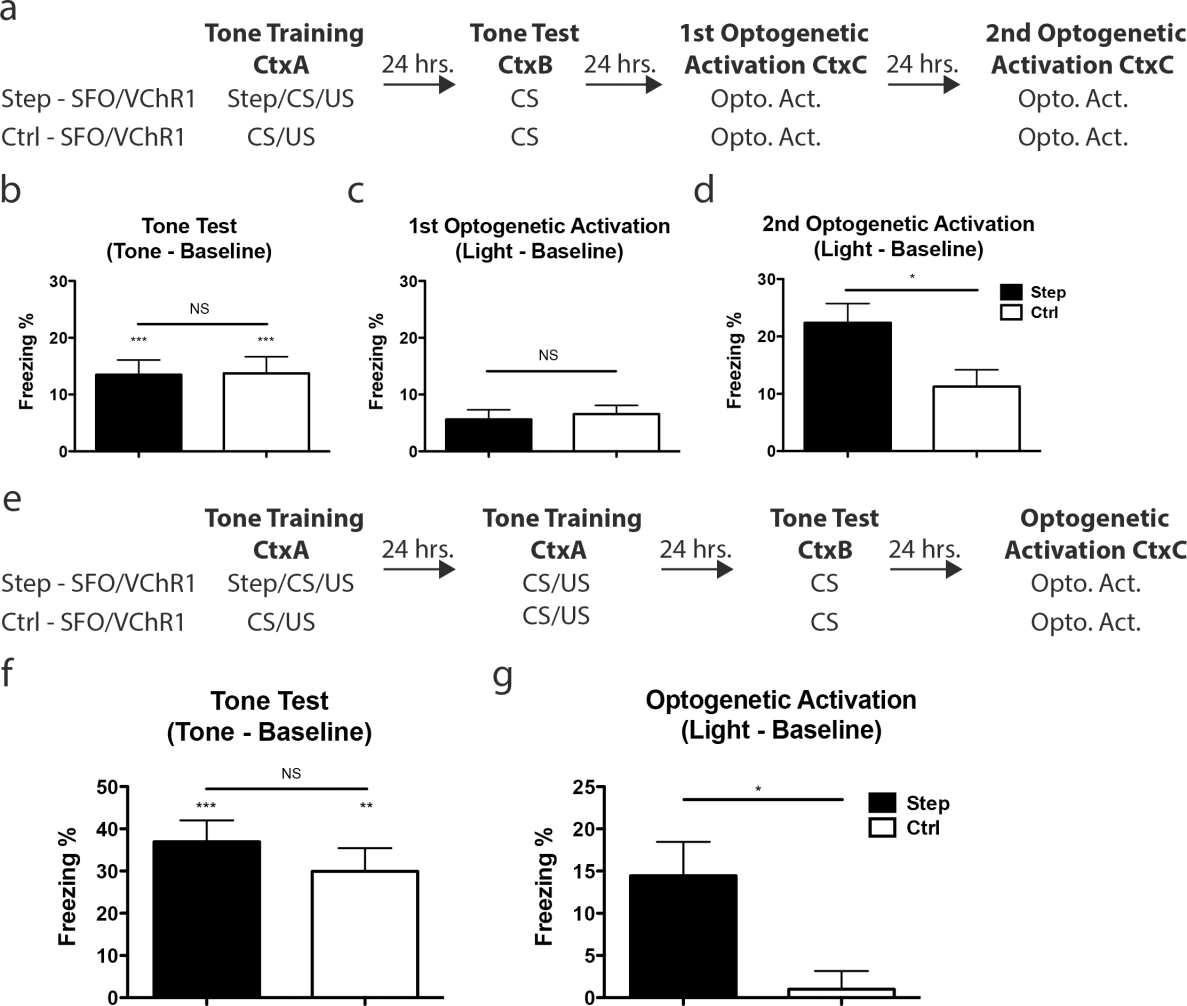
Behavioral controls for multiple training sessions. **(a)** Behavioral design for the two experimental cohorts: Step–SFO/VChR1, and Ctrl–SFO/VChR1. **(b)** There was no difference in freezing during the tone test between Step—SFO/VChR1 and Ctrl—SFO/VChR1 cohorts (unpaired, two-sided t-test, t(37) = 0.07, p > 0.05). Step—SFO/VChR1 (one-sample t-test against 0, t(21) = 5.16, p < 0.001) and Ctrl—SFO/VChR1 (one-sample t-test against 0, t(16) = 4.70, p < 0.001) cohorts both froze above baseline levels. The mean freezing levels were: Step—SFO/VChR1; 13.48 ± 2.61%, n = 22, and Ctrl—SFO/VChR1; 13.74 ± 2.92%, n = 17. **(c)** There was no difference between Step—SFO/VChR1 and Ctrl—SFO/VChR1 cohorts (unpaired, two-sided t-test, t(36) = 0.39, P > 0.05). The mean freezing levels during the 1^st^ optogenetic activation: Step—SFO/VChR1; 5.61 ± 1.72%, n = 22, and Ctrl—SFO/VChR1; 6.55 ± 1.53%, n = 16. **(d)** Step—SFO/VChR1 froze more during the second optogenetic activation than Ctrl—SFO/VChR1 (unpaired, two-sided, t-test t(35) = 2.41, p < 0.05). The mean freezing levels during the 2^nd^ optogenetic activation: Step—SFO/VChR1; 22.38 ± 3.35%, n = 21, and Ctrl—SFO/VChR1; 11.25 ± 2.94%, n = 16. **(e)** Behavioral design for the two experimental cohorts: Step—SFO/VChR1, and Ctrl—SFO/VChR1. **(f)** There was no difference in freezing during the tone test between Step—SFO/VChR1 and Ctrl—SFO/VChR1 cohorts (unpaired, two-sided t-test, (t(9) = 0.93, P > 0.05). Step—SFO/VChR1 (one-sample t-test against 0, t(5) = 7.25, p < 0.001) and Ctrl—SFO/VChR1 (one-sample t-test against 0, t(4) = 5.45, p < 0.01) cohorts both froze above baseline levels. The mean freezing levels during the tone test: Step—SFO/VChR1; 36.93 ± 5.10%, n = 6, and Ctrl—SFO/VChR1; 29.96 ± 5.50%, n = 5. **(g)** Step—SFO/VChR1 froze more during optogenetic activation than Ctrl—SFO/VChR1 (unpaired, two-sided t-test, t(9) = 2.77, p < 0.05). The mean freezing levels: Step—SFO/VChR1; 14.44 ± 4.03%, n = 6, and Ctrl—SFO/VChR1; 1.00 ± 2.15%, n = 5. Error bars are mean ± SEM, * = p < 0.05, ** = p < 0.01, *** = p < 0.001, NS = not significant. See S3 Fig for baseline freezing levels and analysis.

Next, we tested whether the strength of training is critical for the ability of optogenetic activation to trigger recall in the SFO experiments. Thus, Step—SFO/VChR1 and Ctrl—SFO/VChR1 cohorts received a single session (not two sessions as we described before) of AFC paired with or without SFO activation prior to training, respectively (**Fig 5A**). As predicted, both the Step—SFO/VChR1 and Ctrl—SFO/VChR1 cohorts show low levels of freezing during the tone test (**Fig 5B**). Accordingly, optogenetic activation triggered low levels of freezing in both groups (no difference between the Step—SFO/VChR1 and Ctrl—SFO/VChR1 cohorts; **Fig 5C**). Thus, the strength of training (**Fig 5B** compared to **Fig 4B**) is critical for the ability of optogenetic activation to trigger recall in the SFO experiments. Interestingly, a second round of optogenetic activation of the Step—SFO/VChR1 cohort did result in higher levels of freezing than the Ctrl—SFO/VChR1 cohort (**Fig 5D**).

The Step—SFO/VChR1 cohort mentioned in Fig 4 received two days of training and on each day was paired with SFO activation to increase the excitability of SFO/VChR1 expressing neurons prior to AFC. If the allocation of a given memory occurs upon initial exposure, then enhancing excitability on the second day of AFC should not be necessary for memory allocation. To directly test this hypothesis, another cohort of mice was infected with SFO/VChR1 and three weeks later split into two cohorts. The Step—SFO/VChR1 cohort was trained with two trials of AFC separated by 24 hours. However, only the first AFC training was paired with SFO activation (**Fig 5E**), unlike the Step—SFO/VChR1 cohort mentioned in Fig 4 (**Fig 4A)**. The Ctrl—SFO/VChR1 cohort was trained as before with two trials of AFC neither of which was paired with SFO activation (**Fig 5E)**. Analyses of the results of the tone test showed that there was no difference between the two cohorts, and that both cohorts froze above baseline, demonstrating that both groups acquired the AFC memory (**Fig 5F**). Importantly, the Step—SFO/VChR1 cohort showed higher freezing than the Ctrl—SFO/VChR1 cohort during optogenetic activation (**Fig 5G**). This result supports the hypothesis that the AFC memory is allocated in response to the enhanced excitability of the SFO/VChR1 expressing neurons during the first round of training. Subsequent training, whether with SFO activation (**Fig 4A**) or without (**Fig 5E**), simply strengthens a previously allocated memory.

Next, we tested the possibility that SFO activation may act to strengthen the CS (tone), apart from its proposed effect on memory allocation (**Fig 4C**) [20, 21]. Our findings, however, indicate that this is unlikely since both Step—SFO/VChR1 and Ctrl—SFO/VChR1 showed similar freezing levels during tone testing (**Fig 4B**). If SFO activation acted to strengthen the CS (tone), then we would have expected to see higher levels of freezing during the tone test, which we do not. These results also show that SFO activation does not compete with the tone for a share of the total associative strength conferred by the US, as would be expected with overshadowing [22].

### Neuronal excitability in the lateral amygdala and anxiety

**Fig 6:**
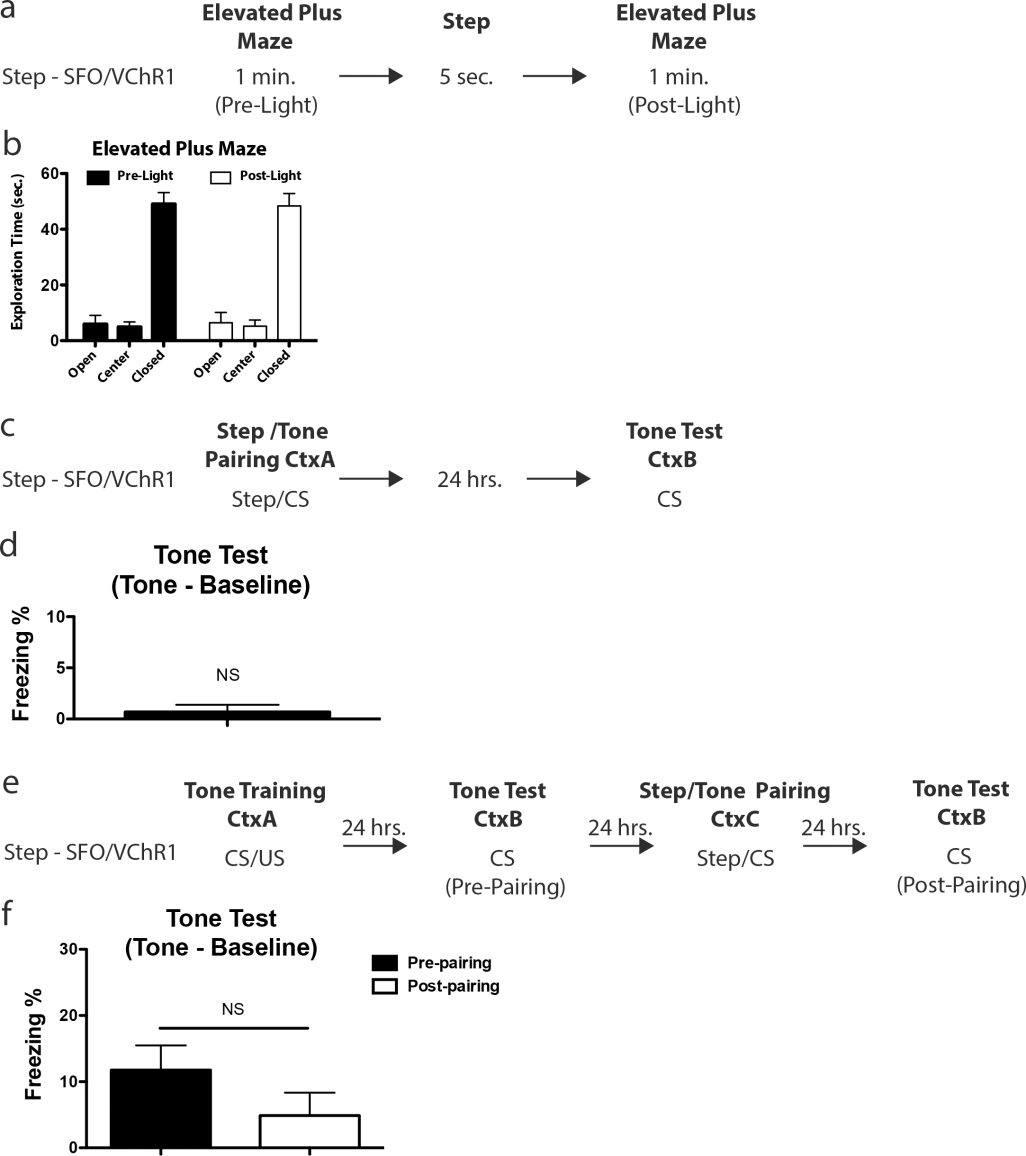
Behavioral controls for excitability induced anxiety and learning. **(a)** Behavioral design for the elevated plus maze (EPM). **(b)** The exploration time in the open, center and closed portions of the EPM was monitored for the minute prior to (Pre-Light) and after (Post-Light) a step in excitability. Two-way ANOVA showed a significant effect of arm (F (2,42) = 109.6, p < 0.001) but no significant interaction (F (2,42) = 0.018, p > 0.05) or effect of light (F(1,42) = 3.7x10^-7^ p > 0.05). The mean exploration times: Pre-Light: Open; 5.96 ± 3.10%, Center; 4.95 ± 1.77%, Closed 49.09 ± 4.06%. Post-Light: Open; 6.43 ± 3.69%, Center; 5.21 ± 2.23%, Closed 48.35 ± 4.46%. **(c)** Behavioral design for the Step/Tone pairing. **(d)** Freezing during tone test for the Step—SFO/VChR1 cohort was not above baseline levels (one sample t-test against 0, t(6) = 1, p > 0.05). The mean freezing during tone test: Step—SFO/VChR1; 0.70 ± 0.70%, n = 7. **e)** Behavioral design for the trained Step/Tone pairing. **(f)** There was no difference in freezing during the tone test between the Step-SFO/VChR1 and Ctrl-SFO/VChR1 cohorts (unpaired, two-sided t-test, t(27) = 1.35, p > 0.05). The mean freezing levels: Pre-Pairing; 11.76 ±3.72%, n = 15 and Post-Pairing; 4.89 ± 3.46%, n = 14 tone test. Error bars are mean ± SEM. See S4 Fig for baseline freezing levels and analysis.

It is possible that increasing excitability by SFO activation, results in increased anxiety, and that this accounts for the higher freezing of the Step—SFO/VChR1 cohort following optogenetic activation. However, our results, and those of others [23], indicate that this possibility is unlikely since both Step—SFO/VChR1 and Ctrl—SFO/VChR1 showed similar freezing scores during tone testing. To test the possibility that SFO activation increases anxiety, a cohort of mice was infected with SFO/VChR1, and then anxiety levels were tested on the elevated plus maze before and after optogenetic stimulation with a 473nm light for 5 seconds (**Fig 6A**). We measured the time in the open arms, center and closed arms, common measures of anxiety [24]. There was no difference in arm preference pre- or post-light activation **(Fig 6B)**, suggesting that there was no increase in anxiety after optogenetic stimulation. This demonstrates that increased anxiety could not account for the higher levels of freezing detected in the Step—SFO/VChR1 cohort during optogenetic activation.

### Neuronal excitability in the lateral amygdala and US strength

SFO activation could conceivably act as an additional US, thus augmenting the effects of foot-shock in our AFC experiments [25]. We addressed this possibility in three different ways. First, our analyses demonstrated that both Step—SFO/VChR1 and Ctrl—SFO/VChR1 showed similar freezing levels during tone testing, demonstrating that SFO activation does not strengthen the US in the cohort with SFO activation immediately before training. Second, we paired SFO activation with a tone. The next day the mice were tone-tested in a different context (**Fig 6C**). Under these conditions, the Step—SFO/VChR1 cohort does not significantly acquire AFC, demonstrating that activation of SFO cannot replace a US (**Fig 6D**). Thirdly, we tested whether the activation of SFO is capable of augmenting the effects of AFC training. To test this, mice were infected with SFO/VChR1 as described above, and trained in AFC 3 weeks later. The animals were then tone tested 24 hours after training in a different context (Pre-Pairing Tone Test). The following day, activation of SFO was paired with a tone. The animals were then tone tested in a different context the next day to determine if the SFO/tone pairing strengthened previous learning of AFC (Post-Pairing Tone Test) (**Fig 6E**). In line with our previous evidence that SFO activation does not act as a US, there was no enhancement in freezing due to SFO/Tone pairing (**Fig 6F**).

## Discussion

Here, optogenetic studies in the lateral amygdala were used to demonstrate that CREB and neuronal excitability regulate which neurons encode an emotional memory. These studies trapped an emotional memory within a subset of lateral amygdala neurons with either CREB or a transient optogenetically-controlled increase in excitability at the time of learning. Subsequent optogenetic activation of this specific subset of lateral amygdala neurons resulted in recall of the memory. Importantly, our control studies showed that these effects were not confounded by anxiety, or alterations in CS or US-strength. Altogether the findings reported here strongly support the hypothesis that CREB regulates memory allocation by modulating neuronal excitability.

Previous results suggested that the transcription factor CREB has a role in neuronal memory allocation [3–7, 9]. Here, we demonstrate that post-training optogenetic activation of neurons infected with a viral CREB is sufficient to elicit recall of an AFC memory **(Fig 2)**. Memory recall, triggered by Channelrhodopsin-2 activation of the neurons with viral CREB, is significantly more robust than that observed after activating a similarly infected population of neurons with a control virus that did not express CREB **(Fig 2C)**. These results suggest that the AFC memory was disproportionally allocated to the neurons with higher CREB, so that Channelrhodopsin-2 activation of these neurons post-training resulted in more robust recall than activation of a similar number of neurons with normal levels of CREB in controls. These results add to growing evidence showing that activation of neurons engaged during learning can lead to memory retrieval [1, 8, 9, 20, 26–31].

Importantly, since we tested the responses to the conditioned stimulus (tone) following AFC, we were able to demonstrate that that the differences in the strength of the optogenetically activated recall were not due to differences in the strength of the memory in the cohorts tested **(Fig 2B)**.

Multiple critical controls were performed that helped us interpret the results described. For example, it was possible that CREB dependent increases in excitability, instead of enhancing memory allocation, enhanced the effects of optogenetic activation, therefore resulting in increased freezing in the mice with CREB virus. To control for this possibility, a cohort of animals was infected with the CREB virus, but was not trained before receiving optogenetic activation. As expected, optogenetic activation of this untrained control cohort did not lead to significant levels of freezing **(Fig 2C)**. This result ruled out the possibility that CREB-dependent increase in excitability enhanced the effects of optogenetic activation, and that this was sufficient to explain the high levels of freezing behavior in the mice with the CREB viruses. However, this control did not rule out the possibility that a CREB-induced increase in excitability together with neuromodulators released following foot shock, and in conjunction with optogenetic activation, might be sufficient to trigger high levels of freezing unrelated to learning. To control for this, a cohort of animals infected with the CREB virus was given foot shock but in a way that did not lead to learning: in these mice the US preceded the CS (unpaired training), a training regimen that is well known not to lead to learning. As expected, these mice did not learn the association between the CS and the US, and optogenetic activation of this control cohort did not lead to significant levels of freezing **(Fig 2C)**. Therefore, the simplest explanation for the results just outlined is that memory for auditory-fear conditioning is preferentially allocated to lateral amygdala neurons infected with a viral CREB vector. Consequently, optogenetic activation of these CREB neurons leads to more robust recall than activation of a similar number of neurons infected with a viral vector that lacked CREB.

Another series of experiments tested the hypothesis that CREB affects memory allocation by increasing neuronal excitability. To test this hypothesis directly, we showed that increasing neuronal excitability in a subset of lateral amygdala neurons right before training increased the probability that these neurons would be involved in memory **(Fig 4)**. This was achieved by specifically enhancing the excitability of a subset of LA neurons during auditory-fear conditioning with an optogenetic tool [17]. Optogenetic activation of these neurons with enhanced excitability was sufficient to elicit recall of an auditory-fear memory **(Fig 4C)**. Importantly, recall in the cohort of mice with optogenetic enhancement in excitability during training was significantly more robust than that measured in another cohort of mice where the enhancement in excitability followed immediately after training **(Fig 4C)**. Crucially, both cohorts of mice showed the same levels of freezing in response to the conditioned stimulus (tone), demonstrating that the differences in recall observed were not due to differences in conditioning **(Fig 4B)**. These results also demonstrated that increases in excitability can bias memory allocation only when they take place during training, and that increases in excitability immediately after learning do not affect neuronal allocation **(Fig 4C)**. Altogether, these results strongly support the hypothesis that increases in neuronal excitability are a mechanism by which higher levels of CREB bias memory to a subset of neurons in a neural circuit.

In agreement with the findings reported here, recently published results [9] with fluorescent in situ hybridization showed that LA neurons expressing a dominant negative potassium channel gene (dnKCNQ2; increases neuronal excitability) or activated with the DREADD (designer receptors exclusively activated by designer drug) system during AFC are more likely to express the immediate early gene *arc* following recall, a result that suggests that these neurons are preferentially involved in encoding the fear memory. Additionally, expressing dnKCNQ2 in LA neurons, or activating LA neurons with either the DREADD (designer receptors exclusively activated by designer drug) system or with optogenetics during AFC [9], increases the strength of the resultant AFC memory, a result that is also consistent with the idea that excitability has a role in neuronal memory allocation. Accordingly, subsequent experiments that activated LA neurons with the DREADD system during AFC were able to elicit recall following reactivation of the same neurons in a novel environment [9]. Recent studies also used IEGs to tag specific populations of neurons for later activation [26–31]. However, these elegant studies were not focused on revealing the mechanisms of memory allocation.

Prior work demonstrates that memory is not stored in every neuron that receives the necessary input to encode it. In fact, it has been shown that only a fraction of the neurons that receive the necessary information to form a memory are integrated into the memory trace [25, 32]. During AFC up to three quarters of LA neurons receive information regarding the CS [33] and the US [34]. However, only about a third of neurons go on to encode the memory [32, 34, 35]. By virally expressing CREB or enhancing neuronal excitability, we were able to bias which neurons encode the memory. CREB affects a number of neuronal processes, such as modulation of intracellular signaling pathways, the expression of plasticity regulated proteins, as well as enhancements of neuronal excitability [7]. Crucially, when we directly enhanced neuronal excitability at the time of training we were also able to bias which neurons encode a memory. This indicates that enhanced neuronal excitability during training and its effect on stimulus-triggered-spiking [5], membrane receptor and channel properties [36], could be an important component of memory allocation. Following the initial memory allocation event, the expression of plasticity related proteins and structural changes needed for long-term memory ensue. Nevertheless, it is likely that increases in excitability alone cannot fully explain memory allocation mechanisms, and that the refinement of a memory trace to a subset of eligible neurons relies on a number of other cellular and circuit mechanisms, such as a lateral inhibition mechanisms in which the most excitable neurons inhibit the formation of the memory in less excitable neurons [1].

In conclusion, the results presented here provide strong support for the hypothesis that neuronal excitability regulates neuronal memory allocation. These findings indicate that one of the ways CREB regulates memory allocation is by modulating neuronal excitability. They also demonstrate that it is possible to not only trap a memory for AFC in a specific subset of LA neurons, but also to trigger recall of that memory by optogenetic activation of those neurons.

## Materials and Methods

### Mice

Adult male, F1 hybrid (C57Bl/6NTac × 129S6/SvEvTac) mice were singly (Fig 2) and group (Figs 4, 5 and 6) housed and maintained on a 12-h light/dark cycle. These mice were used because they show robust and reliable AFC conditioning. Food and water were available ad libitum throughout the experiment. All behavioral manipulations occurred in the light cycle and each cohort of mice was used for a single experiment (except for mice that were tested on the EPM). Behavioral experiments were randomized by an experimenter not performing the behavior or collecting the data. All procedures were approved by the Chancellor’s Animal Research Committee at the University of California at Los Angeles, in accordance with US National Institutes of Health guidelines.

### Lenti viral vectors

Three viral vectors were used in this study pLenti-CREB/ChR2 (CREB/ChR2), pLenti-ChR2 (ChR2) and pLenti-SFO/VChR1 (SFO/VChR1). The vector CREB/ChR2 uses a CMV promoter to drive the expression of a ChR2-mCherry and EGFP-CREB chimera separated by a 2A self-processing viral peptide bridge [37]. A control virus (ChR2) was developed that uses a CMV promoter to drive the expression of a ChR2-mCherry alone. ChR2 is a light gated ion channel derived from Chlamydomonas reinhardtii. When ChR2 absorbs blue light a conformational change occurs that results in the opening of the channel, and consequently in an inward depolarizing current that can trigger action potentials. The vector SFO/VChR1 uses a CMV promoter to drive the expression of a SFO-EYFP and VChR1-mCherry chimera separated by a 2A self-processing viral peptide bridge [37]. SFO is a ChR2 with cysteine 128 mutated to alanine [17]. Unlike ChR2 a pulse of blue light will result in a stable depolarizing step in membrane potential that is maintained following the offset of blue light. VChR1 is a cation-conducting channelrhodopsin from *Volvox carteri* that functions in an analogous way to ChR2 [18]. However unlike ChR2, VChR1 responds maximally to green light allowing it to be used in conjunction with SFO. All pLenti constructs were pseudotyped with VSVg and utilized the CMV promoter to drive gene expression. Prior studies have demonstrated that pLenti-CMV gene expression is primarily found in excitatory vs inhibitory cells at a ratio of ~9:1 a ratio not dissimilar to that of CaMKII expression [38].

### Surgery

All surgeries were done in the Franz Hall animal facility, in accordance with US National Institutes of Health guidelines. Mice of at least 3 months of age were treated with carprofen (5 mg per kg of body weight, subcutaneous), anesthetized with sodium pentobarbital (30–70 mg per kg, intraperitoneal) and placed in a stereotaxic frame. Core body temperature was maintained at 37.5 C with a heating pad. Eyes were coated with a thin layer of ophthalmic ointment to prevent desiccation. The skin above the skull was retracted and two bore holes were drilled in the skull bilaterally above the lateral amygdala (anterior-posterior = –1.4mm, medial-lateral = ±3.3mm from bregma) according to Paxinos and Franklin mouse brain atlas. Cannula (Plastics One, Roanoke, Virginia) were slowly lowered through the bore holes to the correct depth (ventral = – 4.0 mm from bregma). At this point, dental cement was used to fix the cannula in place and seal the wound. Caps were inserted in the cannula in order to prevent debris entering the brain. Mice were then removed from the stereotaxic apparatus and allowed to recover on a water-circulating heating pad. When fully alert, mice were returned to their home cage and placed on a two-week course of antibiotics. The body weight and general condition of each mouse was assessed daily. If any animal demonstrated signs of discomfort, distress, excessive weight loss (<85% of pre-surgery weight) or damaged/clogged their cannula they were removed from the study and humanely euthanized via CO_2_ inhalation. A minority of animals (~5) did demonstrate signs of discomfort, distress, excessive weight loss, likely due to surgically induced brain hemorrhage and/or infection, and were removed from the study and promptly euthanized. Another minority of animals (~15) damaged/clogged their cannula either prior to viral infusion or behavior and were also removed from the study and euthanized.

### Viral infusion

Viral infusion was performed 7 days after surgery to ensure physical recovery. Mice were treated with carprofen (5 mg per kg of body weight, subcutaneous), and anesthetized with sodium pentobarbital (15–35 mg per kg, intraperitoneal). Core body temperature was maintained at 37.5 C with a heating pad. Eyes were coated with a thin layer of ophthalmic ointment to prevent desiccation. Cannula caps were removed and a virus solution (1.0–1.3 μl, bilateral) was delivered to the lateral amygdala (ventral = – 4.8 mm from bregma) at a flow rate of 0.065–0.130 μl per minute through a 22 gauge, 0.8mm projection inner injection cannula (Plastic One, Roanoke, Virginia) attached by polyethylene tubing to Hamilton microsyringes mounted in an infusion pump (Harvard Instruments, Holliston, Massachusetts). The infusion cannula were left in place an additional 10 minutes to ensure diffusion of the vector. Cannula caps were replaced. Mice were allowed to recover on a water-circulating heating pad. When fully alert, they were returned to their home cage. The body weight and general condition of each mouse was assessed daily. Behavioral experiments were performed three weeks following viral infusions to allow viral gene expression levels to plateau.

### Electrophysiology

HEK293T cells were cultured in DMEM medium supplemented with 10% fetal bovine serum, 4mM L-Glutamine and 1% penicillin/streptomycin. Cells were plated onto coverslips and lentivirus was infected 3 days before recording. Photocurrents in HEK cells infected with ChR2, SFO and VChR1 were recorded by conventional whole-cell patch-clamp. The external solution contained [mM]: 135 NaCl, 5 KCl, 10 HEPES, 2 CaCl_2_, 1 MgCl_2_, 30 d-Glucose (pH 7.4). The internal solution contained [mM]: 8 NaCl, 2 KH_2_PO_4_, 2 d-Glucose, 10 HEPES, 130 KMeSO_4_, 4 Mg-ATP, 7 Phosphocreatine Na, 0.3 GTP, 0.5 ADP. Patch pipettes were pulled with micropipette puller model P-97 (Sutter Instrument Co., Novato, California) from borosilicate glass capillary (World Precision Instruments, Inc., Sarasota, Florida) with around 3 M resistance. Cells were visualized with an upright microscope using infrared or epifluorescent illumination, and whole-cell voltage-clamp recordings were made from cells with Multiclamp 700B. Responses were filtered at 2 kHz and digitized at 10 kHz. All data were acquired, stored and analyzed using pClamp 10 (Molecular Devices, Sunnyvale, California). These data are simply a replication of previous work [14, 17, 18].

### Histology and immunohistochemistry

After the behavioral experiments, mice were sacrificed with sodium pentobarbital (140 mg per kg, intraperitoneal) and fixed with a transcardial perfusion with 4% paraformaldehyde in 0.1 M phosphate buffer (pH 7.4, wt/volume). Brains were sliced coronally (40 μm). The cannula tip locations were confirmed at the end of each experiment. Only those mice with validated bilateral cannula placements were included in the analysis. For cfos immunohistochemistry mice were sacrificed 90 minutes following behavior with sodium pentobarbital (140 mg per kg, intraperitoneal) and fixed with a transcardial perfusion with 4% paraformaldehyde in 0.1 M phosphate buffer (pH 7.4, wt/volume). Brains were sliced coronally (40 μm) and prepared for immunocytochemistry using either anti cFos (1:200) rabbit polyclonal antibodies (7963 Abcam, Cambridge, United Kingdom) and an Alexa-568 conjugated anti-rabbit secondary antibody (1:250) (A-11011 Thermo Fisher Scientific, Waltham, Massachusetts), or anti GFP (1:1000) chicken polyclonal antibodies (1020 Aves, Tigard, Oregon) and an Alexa-568 conjugated anti-chicken secondary (1:500) (A11041 Thermo Fisher Scientific) or anti mCherry (1:1000) mouse monoclonal antibody (632532 Clonetech, Mountain View, California) and a Alexa-568 conjugated anti-mouse secondary (1:500) (A-11004 Thermo Fisher Scientific). For visualization of neurons an Alexa-455 conjugated Nissl NeuroTrace (Thermo Fisher Scientific) counter stain was used. Confocal microscopy was used to identify immunoreactive neurons. The lateral amygdala was anatomically defined according to the Paxinos and Franklin atlas [39]. Immunoreactive neurons were counted with a fixed sample window across three sections per animal, in at least three different animals, by an experimenter blind to the treatment condition. Scores were expressed as a percentage of immunoreactive neurons in a defined region (~250um from the tip of the cannula).

### Optogenetic Setup

An optogenetic setup **(Fig 1C)** was designed to bilaterally channel 473 or 532 nm light through fiber optic cables designed to be inserted into the cannula implanted during surgery. The light source consisted of two DPSS lasers (Dragon Lasers, Chang Chun, China) operating at 473 nm and 532 nm delivering TEM00 mode laser beams. The beams are then coupled using a dichroic mirror to make both the beam co-linear and then expanded using a two lens telescope system. The expanded beam is then split in two arms at orthogonal directions using 50:50 beam splitter and then focused by two separate biconvex lenses on to a fiber coupling assembly with xyz adjustments. The optical fiber (200 um multi-mode) from both the arms are aligned using the XYZ adjustments to obtain an equal power output (+/- 2 mW) as measured by power meter held at the other end of the optical fiber. The laser source is modulated using TTL pulses generated from a signal generator (BK precision, Yorba Linda, California). All the optical components including optical fiber are bought from Thorlabs, Newton, New Jersey. The end of the fiber to be inserted into the cannula was reinforced with a steel tube (Small Parts Inc., Logansport Indiana). A Plastics One injector and dummy were modified to allow the fiber to be positioned securely at and appropriate depth during behavior [40].

### Behavior: CREB/ChR2 and ChR2 infected cohorts

AFC (Tone Training) for Trained–CREB/ChR2 and Trained–ChR2 cohorts **(Fig 2A)** entailed the placement of mice in a conditioning chamber, and 1 minute later, the presentation of a tone (2800 Hz, 90 dB, 30 seconds) that co-terminated with a shock (2 sec, 0.7 mA). AFC was tested (Tone Test) 30 minutes after training. Mice were placed in a novel chamber, and 1 minute later, the tone was presented for 1 minute. The index of memory, freezing, was assessed via automated procedures (Med Associates Inc., St. Albans, Vermont). 15 minutes later ChR2 activation was achieved by illuminating the LA of Trained–CREB/ChR2 and Trained–ChR2 cohorts with a 5mW 473nm laser (5Hz, 5ms pulse width) 1 minute after placement in a novel chamber. Activation continued for 1 minute (Optogenetic Activation) and the index of memory, freezing, was assessed via manual scoring procedures by researchers blind to the experimental conditions. The No-Training–CREB/ChR2 cohort was treated identically to the Trained–CREB/ChR2 and Trained–ChR2 cohorts expect no shock was administered during AFC. The Unpaired–CREB/ChR2 cohort was treated identically to the Trained–CREB/ChR2 and Trained–ChR2 cohorts except the shock preceded the tone by 1 minute. Any animal that had a loose or lost head cap, demonstrated signs of sickness or died, were excluded.

### Behavior: SFO/VChR1 infected cohorts

AFC (Tone Training) for Step–SFO/VChR1, Ctrl–SFO/VChR1 and Reversed–SFO/VChR1 cohorts **(Fig 4A)** entailed the placement of mice in a conditioning chamber, and 1 minute later, the presentation of a tone (2800 Hz, 90 dB, 30 seconds) that co-terminated with a shock (3 sec, 0.7 mA). Only the Step–SFO/VChR1 cohort received a 5 second pulse of 5mW 473nm blue light prior to the tone/shock presentation and, thereby, the depolarization of a subset of their neurons during the tone/shock association. The Reversed–SFO/VChR1 cohort received a 5 second pulse of 5mW 473nm blue light 55 seconds after the tone/shock presentation. All three cohorts were trained identically a second time 24 hours later. The mice received two training trials because the manipulations required for SFO activation (bilateral insertion of fiber optics) prior to AFC resulted in reduced AFC learning (Fig 2B compared to Fig 5B). Testing for AFC occurred 24 hours after the second training (Tone Test). Mice were placed in a novel chamber, and 1 minute later, the tone was presented for 1 minute. The index of memory, freezing, was assessed via automated procedures (Med Associates Inc.). 24 hours later VChR1 activation was achieved by illuminating the LA of all three cohorts with a 10mW 532nm laser (10Hz, 10ms pulse width) 1 minute after placement in a novel chamber. Activation continued for 1 minute (Optogenetic Activation). The index of memory, freezing, was assessed via manual scoring procedures by researchers blind to the conditions of the experiment. The Step–SFO/VChR1 and Ctrl–SFO/VChR1 cohorts from **(Fig 5A)** were treated identically to those Fig 4 except they received a single session of tone training and two sessions of optogenetic activation separated by 24 hours. The Step–SFO/VChR1 and Ctrl–SFO/VChR1 cohorts from **(Fig 5E)** were treated identically to those Fig 4 except the Step–SFO/VChR1 cohort did not receive a 5 second pulse of 5mW 473nm blue light immediately prior to the tone/shock presentation on the second day of tone training.

For the elevated plus maze **(Fig 6A)** a cohort of SFO/VChR1 infused animals were placed on an elevated plus maze and allowed to explore for 3 minutes. Each animal then received a 5 second pulse of 5mW 473nm blue light and allowed to explore the elevated plus maze for another 3 minutes. The time spent in the open, center or closed portions of the maze for the minute prior to (Pre-Light) and following (Post-Light) blue light delivery was assessed with automated procedures.

Pairing of step function opsin and tone **(Fig 6C)** entailed the placement of SFO/VChR1 infused animals in a conditioning chamber, and 55 seconds later SFO was activated with a 5 second pulse of 5mW 473nm blue light. The presentation of a tone (2800 Hz, 90 dB, 30 seconds) immediately followed the SFO activation. Testing for AFC occurred 24 hours later. Mice were placed in a novel chamber, and 1 minute later, the tone was presented for 1 minute. Freezing was assessed with automated procedures. These animals were previously tested on the elevated plus maze.

Pairing of step function opsin and tone on previously conditioned animals **(Fig 6E)** entailed the placement of SFO/VChR1 infused animals in a conditioning chamber, and 1 minute later, a tone (2800 Hz, 90 dB, 30 seconds) was presented that co-terminated with a foot-shock (2 sec, 0.7 mA). Testing for AFC occurred 24 hours after training. Mice were placed in a novel chamber, and 1 minute later, the tone was presented for 1 minute (Pre-Pairing Tone Test). 24 hours later mice were placed in a novel chamber, and 55 seconds later SFO was activated with a 5 second pulse of 5mW 473nm blue light. SFO activation was immediately followed by the presentation of a tone (2800 Hz, 90 dB, 30 seconds). Testing for AFC occurred 24 hours later (Post-Pairing Tone Test). Mice were placed in a chamber, and 1 minute later, the tone was presented for 1 minute. Any animal that had a loose or lost head cap, demonstrated signs of sickness or died, were excluded.

### Statistics

A priori knowledge regarding the (n) needed to observe a significant result (in a given behavioral test) was used to determine the sample size. This was subsequently confirmed by observing the variance and significance of each individual experiment. Behavioral results over two standard deviations from the mean were excluded from analysis. One-sample two-sided t-tests against 0 were used to demonstrate statistically significant levels of auditory-fear learning. Unpaired two-sided t-tests are used to determine statistical differences between two cohorts. One-way (baseline subtracted) and two-way (raw data) analysis of variance (ANOVA) were used to determine statistical differences when three or more cohorts were present. Post hoc Tukey’s multiple comparison (one-way) or Bonferroni tests (two-way) were performed to further determine the relationships between cohorts if significance was demonstrated by ANOVA. In the caption of all figures we state which analysis has been used and that error bars are mean ± SEM, * = p < 0.05, ** = p < 0.01, *** = p < 0.001.

## Supporting Information

S1 Fig Optogenetic activation of a CREB allocated memory trace (with baseline freezing values and no excluded data). **(a)** Behavioral design for the four experimental cohorts: Trained—CREB/ChR2, Trained—ChR2, No-Training–CREB/ChR2 and Unpaired—CREB/ChR2. **(b)** A two-way ANOVA demonstrated a difference in freezing between cohorts (F(3,94) = 7.95, p < 0.001), baseline vs tone (F(1,94) = 23.11, p < 0.001) and an interaction between cohorts and baseline vs tone (F(3,94) = 3.04, p < 0.05) during the tone test. Bonferroni’s post hoc test determined that there was a significant difference between baseline vs tone freezing for the Trained—CREB/ChR2 (p < 0.01) and the Trained—ChR2 (p < 0.001) cohorts whereas there was no difference between baseline vs tone for the No-Training–CREB/ChR2 (p > 0.05) and Unpaired—CREB/ChR2 (p > 0.05) cohorts. Mean freezing levels were (baseline / tone): Trained—CREB/ChR2 6.58 ± 2.09%, / 27.12 ± 6.90% n = 11; Trained—ChR2 7.47 ± 2.36% / 29.10 ± 4.93%, n = 17; No-Training—CREB/ChR2 1.21 ± 1.01% / 3.12 ± 2.11%, n = 12; and Unpaired—CREB/ChR2 3.06 ± 1.34% / 11.62 ± 4.89%, n = 11. **(c)** A two-way ANOVA demonstrated a difference in freezing between cohorts (F(3,94) = 11.39, p < 0.001), baseline vs light (F(1,94) = 30.24, p < 0.001) and an interaction between cohorts and baseline vs light (F(3,94) = 6.94, p < 0.001) during the optogenetic activation. Bonferroni’s post hoc test determined that there was a significant difference between baseline vs light freezing for the Trained—CREB/ChR2 (p < 0.001) and the Trained—ChR2 (p < 0.01) cohorts whereas there was no difference between baseline vs light freezing for the No-Training–CREB/ChR2 (p > 0.05) and Unpaired—CREB/ChR2 (p > 0.05) cohorts. Importantly, Bonferroni’s post hoc test also determined that there was a significant difference in freezing to light between the Trained—CREB/ChR2 and the Trained—ChR2 cohorts (p < 0.001), the Trained—CREB/ChR2 and the No-Training–CREB/ChR2 cohorts (p < 0.001), and the Trained—CREB/ChR2 and the Unpaired—CREB/ChR2 cohorts (p < 0.001). Mean freezing levels were (baseline / light): Trained—CREB/ChR2 6.06 ± 3.91% / 48.79 ± 9.21%, n = 11; Trained—ChR2 1.27 ± 0.69% / 20.20 ± 5.87%, n = 17; No-Training—CREB/ChR2 1.81 ± 1.66% / 7.22 ± 3.73%, n = 12; and Unpaired—CREB/ChR2 0.00 ± 0.00% / 5.91 ± 5.09%, n = 11. Error bars are mean ± SEM, ** = p < 0.01, *** = p < 0.001, NS = not significant.
(TIF)

S2 Fig Optogenetic activation of a memory trace allocated by excitability (with baseline freezing values and no excluded data). **(a)** Behavioral design for the three experimental cohorts: Step—SFO/VChR1, Ctrl—SFO/VChR1 and Reversed—SFO/VChR1. **(b)** A two-way ANOVA demonstrated a difference in freezing between baseline vs tone (F(1,96) = 40.96, p < 0.001) but no difference in freezing between cohorts (F(2,96) = 0.87, p > 0.05) and no interaction between cohorts and baseline vs tone (F(2,96) = 1.89, p > 0.05) during the tone test. Bonferroni’s post hoc test determined that there was a significant difference between baseline vs tone freezing for the Step—SFO/VChR1 (p < 0.001), Ctrl—SFO/VChR1 (p < 0.01) and Reversed—SFO/VChR1 (p < 0.01) cohorts. Mean freezing levels were (baseline / tone): Step—SFO/VChR1 33.92 ± 4.24%, / 69.13 ± 5.75% n = 21; Ctrl—SFO/VChR1 40.89 ± 7.59% / 73.67 ± 5.12%, n = 13; and Reversed—SFO/VChR1 35.60 ± 6.87% / 63.47 ± 6.48%, n = 17. **(c)** A two-way ANOVA demonstrated a difference in freezing between cohorts (F(2,96) = 5.56, p < 0.01) and baseline vs light (F(1,96) = 14.19, p < 0.001) but no interaction between cohorts and baseline vs light (F(2,96) = 2.03, p > 0.05) during the optogenetic activation. Bonferroni’s post hoc test determined that there was a significant difference between baseline vs light freezing for the Step—SFO/VChR1 (p < 0.001) cohort whereas there was no difference between baseline vs light freezing for the Ctrl—SFO/VChR1 (p > 0.05) and Reversed—SFO/VChR1 (p > 0.05) cohorts. Importantly, Bonferroni’s post hoc test also determined that there was a significant difference in freezing to light between the Step—SFO/VChR1 and the Ctrl—SFO/VChR1 cohorts (p < 0.01), and the Step—SFO/VChR1 and the Reversed—SFO/VChR1 cohorts (p < 0.01). Mean freezing levels were (baseline / light): Step—SFO/VChR1 5.95 ± 1.43% / 22.14 ± 4.86%, n = 21; Ctrl—SFO/VChR1 3.21 ± 1.65% / 8.21 ± 2.75%, n = 13; Reversed—SFO/VChR1 1.76 ± 0.76% / 8.33 ± 2.49%, n = 17. Error bars are mean ± SEM, ** = p < 0.01, *** = p < 0.001, NS = not significant.
(TIF)

S3 Fig Behavioral controls for multiple training sessions (with baseline freezing values and no excluded data). **(a)** Behavioral design for the two experimental cohorts: Step–SFO/VChR1, and Ctrl–SFO/VChR1. **(b)** A two-way ANOVA demonstrated a difference in freezing between baseline vs tone (F(1,76) = 13.57, p < 0.001) but no difference in freezing between cohorts (F(1,76) = 0.59, p > 0.05) and no interaction between cohorts and baseline vs tone (F(1,76) = 0.02, p > 0.05) during the tone test. Bonferroni’s post hoc test determined that there was a significant difference between baseline vs tone freezing for the Step—SFO/VChR1 (p < 0.01) and Ctrl—SFO/VChR1 (p < 0.05) cohorts. Mean freezing levels were (baseline / tone): Step—SFO/VChR1 9.99 ± 2.83%, / 24.68 ± 4.66% n = 23; Ctrl—SFO/VChR1 7.51 ± 2.62% / 21.25 ± 4.38%, n = 17. **(c)** A two-way ANOVA demonstrated a difference in freezing between baseline vs light (F(1,76) = 17.11, p < 0.001) but no difference in freezing between cohorts (F(1,76) = 0.08, p > 0.05) and no interaction between cohorts and baseline vs light (F(1,76) = 0.04, p > 0.05) during the 1^st^ optogenetic activation. Bonferroni’s post hoc test determined that there was a significant difference between baseline vs light freezing for the Step—SFO/VChR1 (p < 0.01) and Ctrl—SFO/VChR1 (p < 0.05) cohorts. Bonferroni’s post hoc test also determined that there was no significant difference in freezing to light between the Step—SFO/VChR1 and the Ctrl—SFO/VChR1 cohorts (p > 0.05). Mean freezing levels were (baseline / light): Step—SFO/VChR1 2.10 ± 0.59% / 9.85 ± 2.83%, n = 23; Ctrl—SFO/VChR1 1.18 ± 0.37% / 9.69 ± 2.34%, n = 17. **(d)** A two-way ANOVA demonstrated a difference in freezing between baseline vs light (F(1,76) = 34.89, p < 0.001) and a difference in freezing between cohorts (F(1,76) = 4.47, p < 0.05) and no interaction between cohorts and baseline vs light (F(1,76) = 2.63, p > 0.05) during the 2^nd^ optogenetic activation. Bonferroni’s post hoc test determined that there was a significant difference between baseline vs light freezing for the Step—SFO/VChR1 (p < 0.001) and Ctrl—SFO/VChR1 (p < 0.05) cohorts. Importantly, Bonferroni’s post hoc test also determined that there was a significant difference in freezing to light between the Step—SFO/VChR1 and the Ctrl—SFO/VChR1 cohorts (p < 0.05). Mean freezing levels were (baseline / light): Step—SFO/VChR1 6.52 ± 1.42% / 29.78 ± 4.41%, n = 23; Ctrl—SFO/VChR1 5.00 ± 1.46% / 18.23 ± 3.16%, n = 17. **(e)** Behavioral design for the two experimental cohorts: Step—SFO/VChR1, and Ctrl—SFO/VChR1. **(f)** A two-way ANOVA demonstrated a difference in freezing between baseline vs tone (F(1,18) = 27.86, p < 0.001) but no difference in freezing between cohorts (F(1,18) = 0.09, p > 0.05) and no interaction between cohorts and baseline vs tone (F(1,18) = 0.01, p > 0.05) during the tone test. Bonferroni’s post hoc test determined that there was a significant difference between baseline vs tone freezing for the Step—SFO/VChR1 (p < 0.01) and Ctrl—SFO/VChR1 (p < 0.01) cohorts. Mean freezing levels were (baseline / tone): Step—SFO/VChR1 6.38 ± 4.75%, / 42.68 ± 9.69% n = 6; Ctrl—SFO/VChR1 9.06 ± 3.16% / 44.05 ± 6.25%, n = 5. **(g)** A two-way ANOVA demonstrated a difference in freezing between baseline vs light (F(1,18) = 5.45, p < 0.05) but no difference in freezing between cohorts (F(1,18) = 2.04, p > 0.05) and no interaction between cohorts and baseline vs light (F(1,18) = 4.13, p > 0.05) during optogenetic activation. Bonferroni’s post hoc test determined that there was a significant difference between baseline vs light freezing for the Step—SFO/VChR1 (p < 0.01) but not the Ctrl—SFO/VChR1 (p > 0.05) cohorts. Importantly, bonferroni’s post hoc test also determined that there was a significant difference in freezing to light between the Step—SFO/VChR1 and the Ctrl—SFO/VChR1 cohorts (p < 0.05). Mean freezing levels were (baseline / light): Step—SFO/VChR1 5.00 ± 2.30% / 19.44 ± 4.89%, n = 6; Ctrl—SFO/VChR1 7 ± 2.22% / 8.00 ± 2.77%, n = 5. Error bars are mean ± SEM, * = p < 0.05, ** = p < 0.01, *** = p < 0.001, NS = not significant.
(TIF)

S4 Fig Behavioral controls for excitability induced anxiety and learning (with baseline freezing values and no excluded data). **(a)** Behavioral design for the elevated plus maze (EPM). **(b)** The exploration time in the open, center and closed portions of the EPM was monitored for the minute prior to (Pre-Light) and after (Post-Light) a step in excitability. Two-way ANOVA showed a significant effect of arm (F (2,42) = 109.6, p < 0.001) but no significant interaction (F (2,42) = 0.018, p > 0.05) or effect of light (F(1,42) = 3.7x10^-7^ p > 0.05). The mean exploration times: Pre-Light: Open; 5.96 ± 3.10%, Center; 4.95 ± 1.77%, Closed 49.09 ± 4.06%. Post-Light: Open; 6.43 ± 3.69%, Center; 5.21 ± 2.23%, Closed 48.35 ± 4.46%. **(c)** Behavioral design for the Step/Tone pairing. **(d)** There was no difference in freezing during the tone test between the baseline and tone (unpaired, two-sided t-test, t(7) = -1.33, p > 0.05). The mean freezing levels (baseline / tone): 0.00 ± 0.00% / 2.35 ± 1.76%, n = 8 tone test. **e)** Behavioral design for the trained Step/Tone pairing. **(f)** A two-way ANOVA demonstrated no difference in freezing between baseline vs tone (F(1,56) = 2.68, p > 0.05), cohorts (F(1,56) = 0.946, p > 0.05) and no interaction between cohorts and baseline vs tone (F(1,56) = 1.10, p > 0.05) during the tone test. Bonferroni’s post hoc test determined that there was no significant difference between tone freezing for the Step—SFO/VChR1 and Ctrl—SFO/VChR1 (p > 0.05) cohorts. Mean freezing levels were (baseline / tone): Step—SFO/VChR1 13.22 ± 3.49%, / 22.06 ± 4.77% n = 15; Ctrl—SFO/VChR1 24.97 ± 5.12% / 24.64 ± 3.92%, n = 5. Error bars are mean ± SEM, NS = not significant.
(TIF)
