# Supplementary figures and images for: Molecular and Cellular Mechanisms for Trapping and Activating Emotional Memories
Source: PLoS One. 2016 Aug 31;11(8):e0161655. doi: 10.1371/journal.pone.0161655 (PMC5007047; doi:10.1371/journal.pone.0161655)

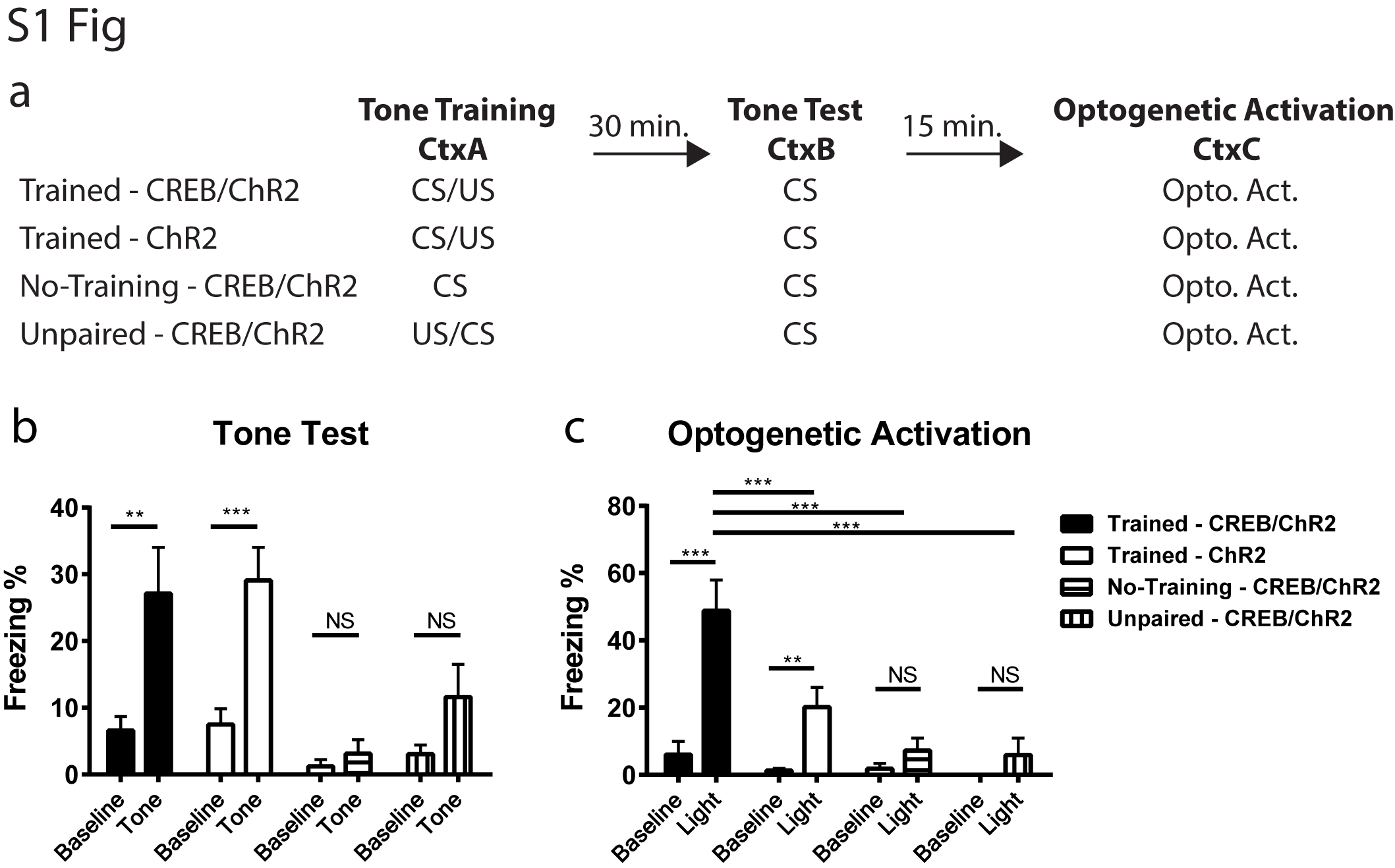

Supplement: S1 Fig — (a) Behavioral design for the four experimental cohorts: Trained—CREB/ChR2, Trained—ChR2, No-Training–CREB/ChR2 and Unpaired—CREB/ChR2. (b) A two-way ANOVA demonstrated a difference in freezing between cohorts (F(3,94) = 7.95, p < 0.001), baseline vs tone (F(1,94) = 23.11, p < 0.001) and an interaction between cohorts and baseline vs tone (F(3,94) = 3.04, p < 0.05) during the tone test. Bonferroni’s post hoc test determined that there was a significant difference between baseline vs tone freezing for the Trained—CREB/ChR2 (p < 0.01) and the Trained—ChR2 (p < 0.001) cohorts whereas there was no difference between baseline vs tone for the No-Training–CREB/ChR2 (p > 0.05) and Unpaired—CREB/ChR2 (p > 0.05) cohorts. Mean freezing levels were (baseline / tone): Trained—CREB/ChR2 6.58 ± 2.09%, / 27.12 ± 6.90% n = 11; Trained—ChR2 7.47 ± 2.36% / 29.10 ± 4.93%, n = 17; No-Training—CREB/ChR2 1.21 ± 1.01% / 3.12 ± 2.11%, n = 12; and Unpaired—CREB/ChR2 3.06 ± 1.34% / 11.62 ± 4.89%, n = 11. (c) A two-way ANOVA demonstrated a difference in freezing between cohorts (F(3,94) = 11.39, p < 0.001), baseline vs light (F(1,94) = 30.24, p < 0.001) and an interaction between cohorts and baseline vs light (F(3,94) = 6.94, p < 0.001) during the optogenetic activation. Bonferroni’s post hoc test determined that there was a significant difference between baseline vs light freezing for the Trained—CREB/ChR2 (p < 0.001) and the Trained—ChR2 (p < 0.01) cohorts whereas there was no difference between baseline vs light freezing for the No-Training–CREB/ChR2 (p > 0.05) and Unpaired—CREB/ChR2 (p > 0.05) cohorts. Importantly, Bonferroni’s post hoc test also determined that there was a significant difference in freezing to light between the Trained—CREB/ChR2 and the Trained—ChR2 cohorts (p < 0.001), the Trained—CREB/ChR2 and the No-Training–CREB/ChR2 cohorts (p < 0.001), and the Trained—CREB/ChR2 and the Unpaired—CREB/ChR2 cohorts (p < 0.001). Mean freezing levels were (baseline / light): Trained—CREB [file pone.0161655.s001.tif]

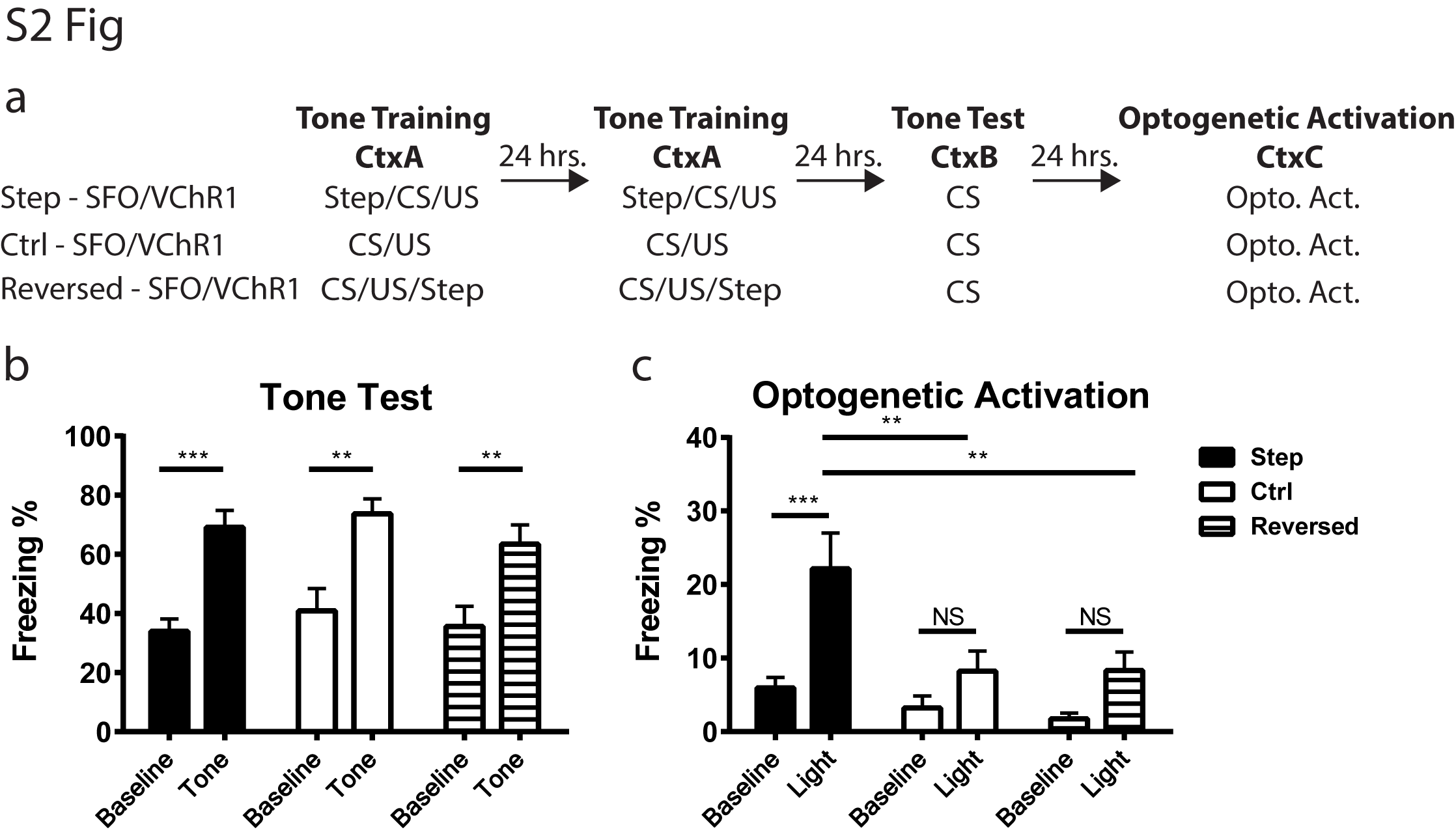

Supplement: S2 Fig — (a) Behavioral design for the three experimental cohorts: Step—SFO/VChR1, Ctrl—SFO/VChR1 and Reversed—SFO/VChR1. (b) A two-way ANOVA demonstrated a difference in freezing between baseline vs tone (F(1,96) = 40.96, p < 0.001) but no difference in freezing between cohorts (F(2,96) = 0.87, p > 0.05) and no interaction between cohorts and baseline vs tone (F(2,96) = 1.89, p > 0.05) during the tone test. Bonferroni’s post hoc test determined that there was a significant difference between baseline vs tone freezing for the Step—SFO/VChR1 (p < 0.001), Ctrl—SFO/VChR1 (p < 0.01) and Reversed—SFO/VChR1 (p < 0.01) cohorts. Mean freezing levels were (baseline / tone): Step—SFO/VChR1 33.92 ± 4.24%, / 69.13 ± 5.75% n = 21; Ctrl—SFO/VChR1 40.89 ± 7.59% / 73.67 ± 5.12%, n = 13; and Reversed—SFO/VChR1 35.60 ± 6.87% / 63.47 ± 6.48%, n = 17. (c) A two-way ANOVA demonstrated a difference in freezing between cohorts (F(2,96) = 5.56, p < 0.01) and baseline vs light (F(1,96) = 14.19, p < 0.001) but no interaction between cohorts and baseline vs light (F(2,96) = 2.03, p > 0.05) during the optogenetic activation. Bonferroni’s post hoc test determined that there was a significant difference between baseline vs light freezing for the Step—SFO/VChR1 (p < 0.001) cohort whereas there was no difference between baseline vs light freezing for the Ctrl—SFO/VChR1 (p > 0.05) and Reversed—SFO/VChR1 (p > 0.05) cohorts. Importantly, Bonferroni’s post hoc test also determined that there was a significant difference in freezing to light between the Step—SFO/VChR1 and the Ctrl—SFO/VChR1 cohorts (p < 0.01), and the Step—SFO/VChR1 and the Reversed—SFO/VChR1 cohorts (p < 0.01). Mean freezing levels were (baseline / light): Step—SFO/VChR1 5.95 ± 1.43% / 22.14 ± 4.86%, n = 21; Ctrl—SFO/VChR1 3.21 ± 1.65% / 8.21 ± 2.75%, n = 13; Reversed—SFO/VChR1 1.76 ± 0.76% / 8.33 ± 2.49%, n = 17. Error bars are mean ± SEM, ** = p < 0.01, *** = p < 0.001, NS = not significant. (TIF) [file pone.0161655.s002.tif]

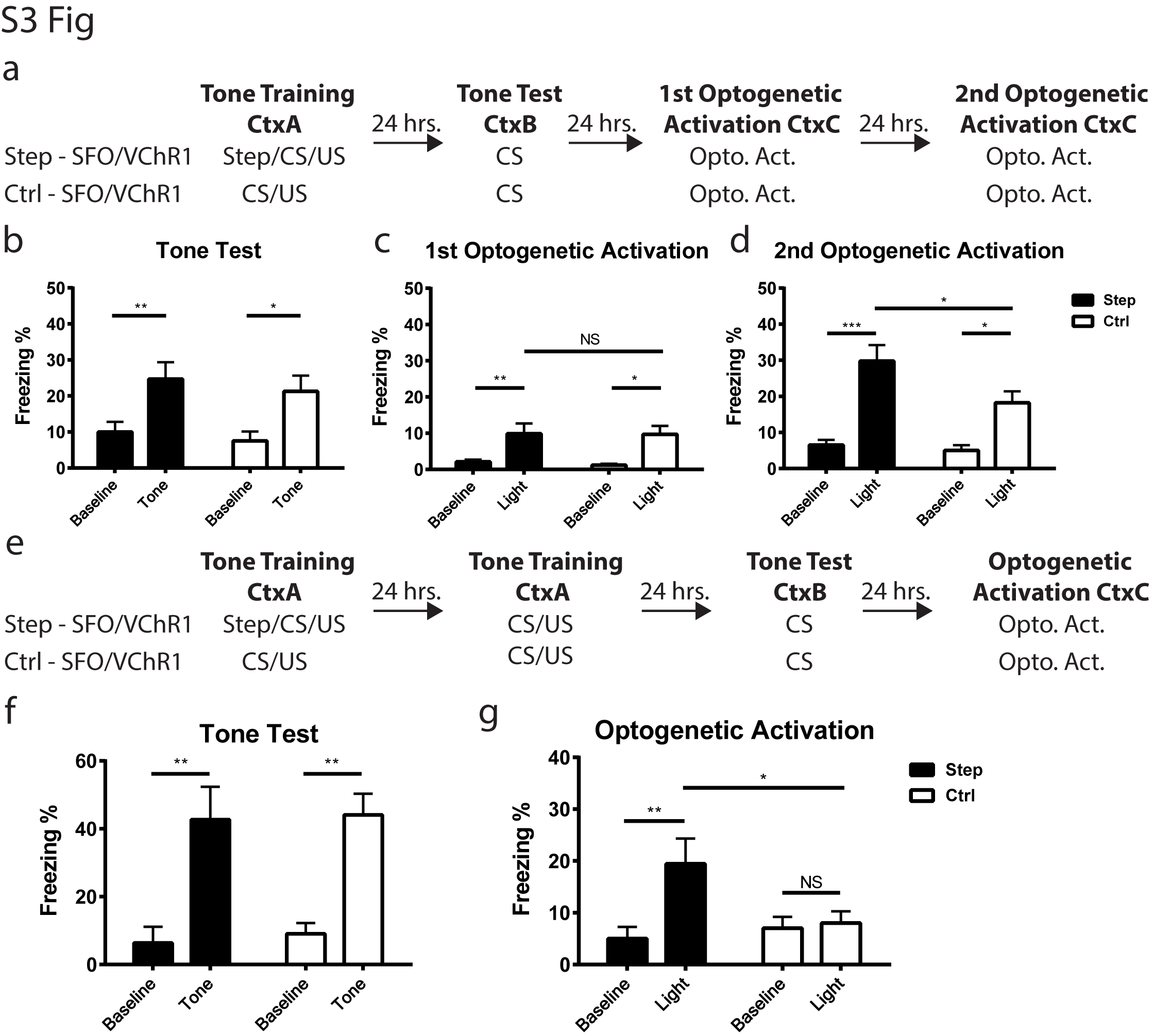

Supplement: S3 Fig — (a) Behavioral design for the two experimental cohorts: Step–SFO/VChR1, and Ctrl–SFO/VChR1. (b) A two-way ANOVA demonstrated a difference in freezing between baseline vs tone (F(1,76) = 13.57, p < 0.001) but no difference in freezing between cohorts (F(1,76) = 0.59, p > 0.05) and no interaction between cohorts and baseline vs tone (F(1,76) = 0.02, p > 0.05) during the tone test. Bonferroni’s post hoc test determined that there was a significant difference between baseline vs tone freezing for the Step—SFO/VChR1 (p < 0.01) and Ctrl—SFO/VChR1 (p < 0.05) cohorts. Mean freezing levels were (baseline / tone): Step—SFO/VChR1 9.99 ± 2.83%, / 24.68 ± 4.66% n = 23; Ctrl—SFO/VChR1 7.51 ± 2.62% / 21.25 ± 4.38%, n = 17. (c) A two-way ANOVA demonstrated a difference in freezing between baseline vs light (F(1,76) = 17.11, p < 0.001) but no difference in freezing between cohorts (F(1,76) = 0.08, p > 0.05) and no interaction between cohorts and baseline vs light (F(1,76) = 0.04, p > 0.05) during the 1st optogenetic activation. Bonferroni’s post hoc test determined that there was a significant difference between baseline vs light freezing for the Step—SFO/VChR1 (p < 0.01) and Ctrl—SFO/VChR1 (p < 0.05) cohorts. Bonferroni’s post hoc test also determined that there was no significant difference in freezing to light between the Step—SFO/VChR1 and the Ctrl—SFO/VChR1 cohorts (p > 0.05). Mean freezing levels were (baseline / light): Step—SFO/VChR1 2.10 ± 0.59% / 9.85 ± 2.83%, n = 23; Ctrl—SFO/VChR1 1.18 ± 0.37% / 9.69 ± 2.34%, n = 17. (d) A two-way ANOVA demonstrated a difference in freezing between baseline vs light (F(1,76) = 34.89, p < 0.001) and a difference in freezing between cohorts (F(1,76) = 4.47, p < 0.05) and no interaction between cohorts and baseline vs light (F(1,76) = 2.63, p > 0.05) during the 2nd optogenetic activation. Bonferroni’s post hoc test determined that there was a significant difference between baseline vs light freezing for the Step—SFO/VChR1 (p < 0.001) and Ct [file pone.0161655.s003.tif]

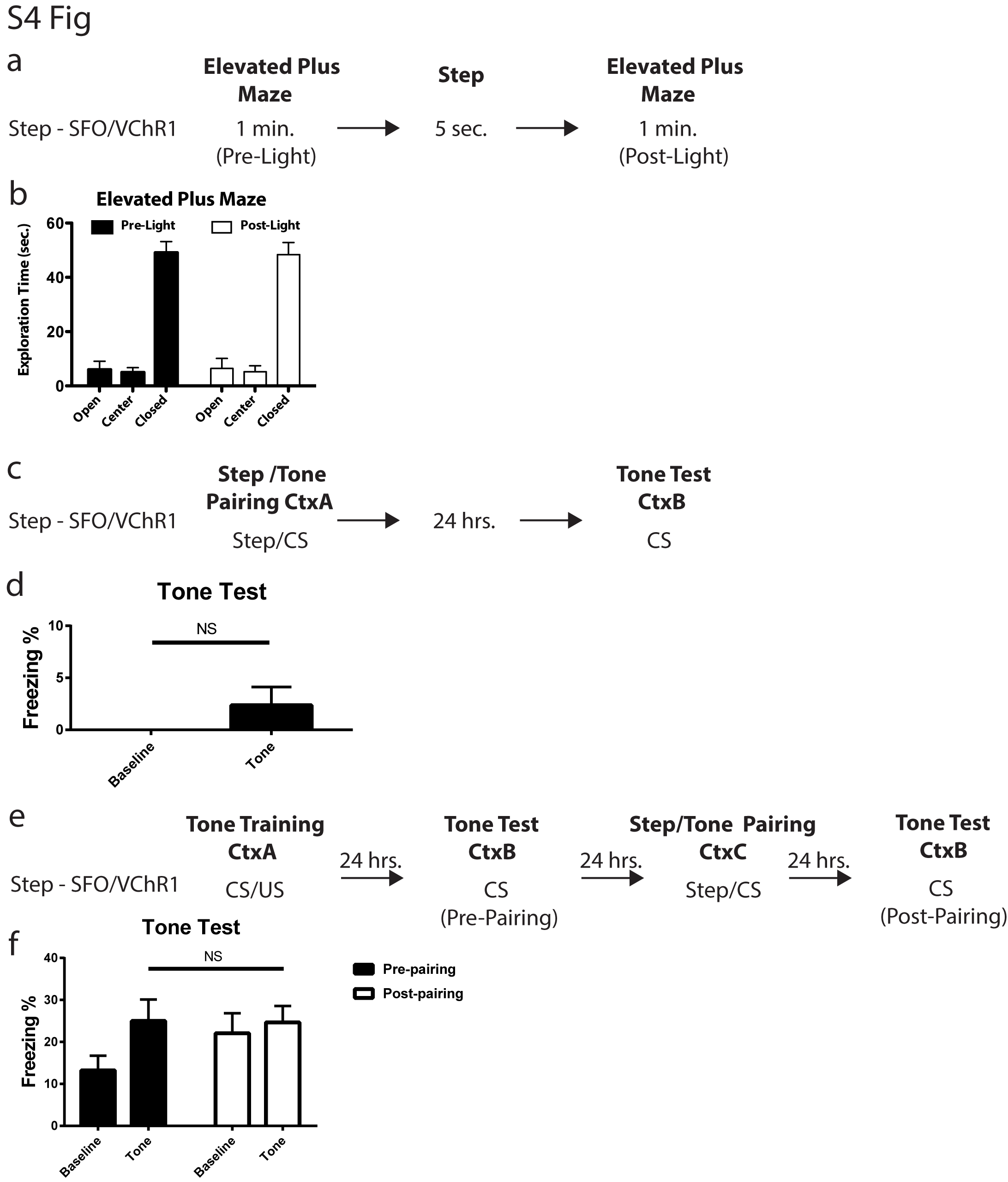

Supplement: S4 Fig — (a) Behavioral design for the elevated plus maze (EPM). (b) The exploration time in the open, center and closed portions of the EPM was monitored for the minute prior to (Pre-Light) and after (Post-Light) a step in excitability. Two-way ANOVA showed a significant effect of arm (F (2,42) = 109.6, p < 0.001) but no significant interaction (F (2,42) = 0.018, p > 0.05) or effect of light (F(1,42) = 3.7x10^-7^ p > 0.05). The mean exploration times: Pre-Light: Open; 5.96 ± 3.10%, Center; 4.95 ± 1.77%, Closed 49.09 ± 4.06%. Post-Light: Open; 6.43 ± 3.69%, Center; 5.21 ± 2.23%, Closed 48.35 ± 4.46%. (c) Behavioral design for the Step/Tone pairing. (d) There was no difference in freezing during the tone test between the baseline and tone (unpaired, two-sided t-test, t(7) = -1.33, p > 0.05). The mean freezing levels (baseline / tone): 0.00 ± 0.00% / 2.35 ± 1.76%, n = 8 tone test. e) Behavioral design for the trained Step/Tone pairing. (f) A two-way ANOVA demonstrated no difference in freezing between baseline vs tone (F(1,56) = 2.68, p > 0.05), cohorts (F(1,56) = 0.946, p > 0.05) and no interaction between cohorts and baseline vs tone (F(1,56) = 1.10, p > 0.05) during the tone test. Bonferroni’s post hoc test determined that there was no significant difference between tone freezing for the Step—SFO/VChR1 and Ctrl—SFO/VChR1 (p > 0.05) cohorts. Mean freezing levels were (baseline / tone): Step—SFO/VChR1 13.22 ± 3.49%, / 22.06 ± 4.77% n = 15; Ctrl—SFO/VChR1 24.97 ± 5.12% / 24.64 ± 3.92%, n = 5. Error bars are mean ± SEM, NS = not significant. (TIF) [file pone.0161655.s004.tif]
